# Respiratory Rehabilitation and Decannulation in Adults with Prolonged Mechanical Ventilation After Tracheostomy: A Narrative Review

**DOI:** 10.3390/healthcare14121804

**Published:** 2026-06-22

**Authors:** Jun Zhang, Xi Zhao, Ming Fen Tao, Hong Mei Zeng, Li Ping Yuan, Emmanuel Mensah, Shuoshuo Wei, Lingling Pan, Lei Zha

**Affiliations:** 1 Department of Pulmonary and Critical Care Medicine, The First Affiliated Hospital of Wannan Medical University (Yijishan Hospital), Wuhu 241001, China; zhangjun1@yjsyy.com (J.Z.); cenghongmei@yjsyy.com (H.M.Z.); emmanuelmensahx@gmail.com (E.M.); weishuoshuo@yjsyy.com (S.W.); 2 Emergency Surgery Department, The First Affiliated Hospital of Wannan Medical University (Yijishan Hospital), Wuhu 241001, China; zhaoxi@yjsyy.com; 3 Nursing Department, The First Affiliated Hospital of Wannan Medical University (Yijishan Hospital), Wuhu 241001, Chinayuanliping@yjsyy.com (L.P.Y.); 4 Internal Medicine Unit, Takoradi Hospital, Takoradi P.O. Box TD7, Ghana; 5 Cardiology Department, The First Affiliated Hospital of Wannan Medical University (Yijishan Hospital), Wuhu 241000, China; panlingling@yjsyy.com

**Keywords:** prolonged mechanical ventilation, tracheostomy, decannulation, respiratory rehabilitation, inspiratory muscle training, weaning, multidisciplinary care

## Abstract

**Highlights:**

**What are the main findings?**

- Respiratory rehabilitation in tracheostomized adults with prolonged mechanical ventilation may benefit from integration of six evidence-based intervention domains: inspiratory muscle training at ≥50% of maximal inspiratory pressure, early exercise and nutritional optimisation, sedation management guided by the ABCDEF bundle, speaking valve use, airway complication management, and ventilator mode optimisation—each of which directly builds the physiological prerequisites for successful decannulation.
- Decannulation readiness is commonly assessed using five structured domains by a multidisciplinary team—neurological readiness, secretion clearance (suctioning ≤ 4 times/24 h), cough efficacy (peak cough flow > 160 L/min), instrumental swallowing assessment, and upper airway patency confirmed by fiberoptic bronchoscopy. Reported decannulation rates vary widely across settings, ranging from 22% in some general ICU cohorts to 40–70% in specialised weaning centres, reflecting the influence of institutional protocols, multidisciplinary team structures, and resource availability alongside patient physiology.

**What are the implications of the main findings?**

- Clinicians and multidisciplinary teams may consider structured, protocol-driven decannulation pathways, including the stepwise algorithm presented in this review, to close the gap between ventilator liberation and successful decannulation, with particular attention to expiratory muscle training and speaking valve integration as underutilised components of the rehabilitation pathway.
- Treatment success in this population must be redefined beyond physiological endpoints to encompass patient-reported outcomes including voice restoration, psychological well-being, and social reintegration; furthermore, adapted rehabilitation protocols for low- and middle-income country settings represent an urgent global health research priority.

**Abstract:**

**Background**: Patients with prolonged mechanical ventilation (PMV) frequently require tracheostomy due to failure to wean, yet the pathway from ventilator dependence to successful decannulation remains complex and poorly standardised. Comprehensive respiratory rehabilitation is recognised as a core strategy for improving decannulation outcomes, but no unified, evidence-based guidelines currently exist for this population. This review addresses that gap by synthesising current evidence on respiratory rehabilitation and decannulation strategies for tracheostomized PMV patients. **Methods**: A narrative review was conducted through a systematic search of PubMed/MEDLINE covering publications indexed from May 2019 to February 2026, supplemented by targeted searches of Embase and the Cochrane Library. The search combined free-text keywords and Medical Subject Headings (MeSH) terms across eight search string combinations. Following title and abstract screening of 830 deduplicated records, 51 studies met eligibility criteria and were included in the final narrative synthesis. **Results**: Six core rehabilitation intervention domains were identified: respiratory muscle training, physical rehabilitation and nutritional optimisation, sedation and delirium management, speaking valve use, airway complication management, and ventilator mode optimisation. High-intensity inspiratory muscle training at no less than 50% of maximal inspiratory pressure is currently supported by the strongest available evidence among the interventions reviewed, although this threshold derives primarily from general ICU populations and has not been specifically validated in heterogeneous tracheostomized PMV cohorts. Decannulation readiness assessment may benefit from evaluating five core domains—neurological readiness, secretion management capacity (suctioning ≤ 4 times/24 h), cough efficacy (peak cough flow > 160 L/min), safe swallowing confirmed by instrumental assessment, and upper airway patency confirmed by fiberoptic bronchoscopy—using a structured multidisciplinary framework. **Conclusions**: Successful decannulation in tracheostomized PMV patients requires integration of evidence-based rehabilitation interventions, structured multidisciplinary assessment, and a patient-centred outcome framework that extends beyond physiological endpoints to encompass voice restoration, psychological well-being, and social reintegration. Significant evidence gaps remain—particularly for expiratory muscle training, population-specific decannulation protocols, and adapted rehabilitation models for resource-limited settings—representing priority areas for future research.

## 1. Introduction

Prolonged mechanical ventilation (PMV) represents a major clinical and economic burden in critical care medicine. Definitions vary widely, ranging from as little as 5 h in surgical populations to up to one year in chronic respiratory failure. A consensus conference convened by the US Association for Medical Direction of Respiratory Care defined PMV as mechanical ventilation lasting ≥21 consecutive days for ≥6 h per day [1]. However, given that average mechanical ventilation duration is typically 4–5 days, a threshold of ≥7 days has been proposed as more clinically meaningful. Using the Joint Task Force definition, PMV is defined as the need for more than 7 days of weaning after the first spontaneous breathing trial (SBT) [2].

Regardless of definition, PMV affects 5–10% of ICU patients, with 10–34% ultimately requiring tracheostomy due to failure to wean [3,4]. The clinical and economic consequences are substantial. Approximately 29% of patients receiving PMV die during hospital admission, while only around 50% achieve successful weaning; one-year direct healthcare costs reach $306,135 per patient—more than three times those ventilated for ≤21 days [4,5]. For patients unable to achieve timely weaning, tracheostomy provides important advantages, including reduced work of breathing, lower sedation requirements, decreased pulmonary infection risk, and a platform for active rehabilitation. However, tracheostomy is associated with complications: 7% of patients experience an adverse event, and 19.7% do not survive to hospital discharge [6].

Respiratory rehabilitation is central to improving outcomes in this population. Evidence demonstrates that targeted interventions can enhance diaphragmatic function, facilitate weaning, shorten hospital stay, and improve survival [7,8,9]. While previous work has examined individual components of PMV management—including weaning strategies, tracheostomy care, and the role of physiotherapy in ventilator liberation [3]—no synthesis has integrated respiratory rehabilitation interventions, decannulation assessment criteria, multidisciplinary management strategies, and influencing factors specifically for tracheostomized PMV patients [10]. The absence of unified, evidence-based guidance for this population remains a critical gap [11]. This review addresses that gap by synthesising current evidence across the rehabilitation-to-decannulation pathway. The aim is to provide a structured, clinically applicable framework to support multidisciplinary teams in optimising respiratory rehabilitation and achieving successful decannulation in adults with PMV following tracheostomy.

## 2. Materials and Methods

### 2.1. Study Design and Rationale

This review was designed as a narrative synthesis of contemporary evidence on respiratory rehabilitation and decannulation in adults with PMV following tracheostomy. It is a narrative review, not a systematic review or meta-analysis. A narrative review methodology was selected because the topic spans heterogeneous domains—including respiratory physiology, rehabilitation interventions, multidisciplinary care models, psychosocial factors, and health system considerations—that preclude the methodological uniformity required for quantitative meta-analysis. Although structured elements were incorporated—including a defined search strategy, eligibility criteria, and a screening process—to enhance transparency and reproducibility, the methodology does not constitute a formal systematic review. The reported number of included studies reflects the outcome of this structured narrative approach. No formal quantitative synthesis was performed, and the absence of complete dual independent screening from the outset and formal risk-of-bias assessment distinguishes this work from a systematic review. The review was conducted in accordance with the principles of the Scale for the Assessment of Narrative Review Articles (SANRA) and the Synthesis Without Meta-analysis (SWiM) reporting framework.

### 2.2. Protocol and Transparency

A review protocol outlining the research question, eligibility criteria, and thematic synthesis plan was developed prior to the literature search. Given the narrative design, prospective registration in PROSPERO was not performed, as this registry is primarily intended for systematic reviews and meta-analyses. All methodological decisions are reported transparently to facilitate reproducibility and critical appraisal.

### 2.3. Literature Search Strategy

A structured search of PubMed/MEDLINE was conducted for publications indexed between May 2019 and February 2026. The May 2019 start date was selected for three reasons: first, several key clinical guidelines and consensus statements directly relevant to tracheostomy rehabilitation and PMV management were published from 2019 onwards, including updates to AARC, ESPEN, and ASPEN guidance; second, the post-2019 period represents a rapid expansion of research in respiratory muscle training, speaking valve use, sedation liberation protocols, and structured decannulation frameworks; and third, foundational pre-2019 studies in this area are already well synthesised in prior reviews, which are cited as background where relevant in this manuscript. The final search date was 15 February 2026.

The search combined free-text keywords—including “prolonged mechanical ventilation,” “tracheostomy,” “tracheotomy,” “decannulation,” “rehabilitation,” “tracheostomy weaning,” and “tracheotomy weaning”—with Medical Subject Headings (MeSH) terms in eight search string combinations covering tracheostomy, mechanical ventilation, rehabilitation, and weaning. Reference lists of included studies were manually reviewed to identify additional sources. A targeted supplementary search of Embase and the Cochrane Library was subsequently performed and did not identify additional high-quality studies that materially altered the conclusions of this review. The eight search string combinations reflected variations in keyword ordering and Boolean operators; the full combined search string is provided in Appendix A.

Grey literature—including professional society clinical practice guidelines, expert consensus statements, and international health organisation reports—was intentionally included where directly relevant to tracheostomy rehabilitation or decannulation practice. Guidelines and consensus documents were eligible for inclusion if they were published by recognised professional societies including AARC, ATS, ERS, ESPEN, ASPEN, FICM, and ICS; addressed adult tracheostomy care, prolonged mechanical ventilation management, or decannulation practice; and fell within the defined search window. Guideline identification combined PubMed searching using the publication type filter [pt guideline] with manual review of relevant professional society websites. The full PubMed search string is provided in Appendix A.

### 2.4. Eligibility Criteria

Studies were eligible for inclusion if they enrolled adult patients aged ≥ 18 years with PMV who had undergone tracheostomy or tracheotomy; employed study designs including randomised controlled trials, observational studies, systematic reviews, clinical practice guidelines, or expert consensus statements; were published in English or Chinese; and fell within the defined search window of May 2019 to February 2026. Studies were excluded if they focused exclusively on patients aged under 18 years, were published before May 2019, included fewer than two participants, or were not relevant to the clinical scope of this review as determined by full-text assessment.

### 2.5. Study Selection

**Figure 1:**
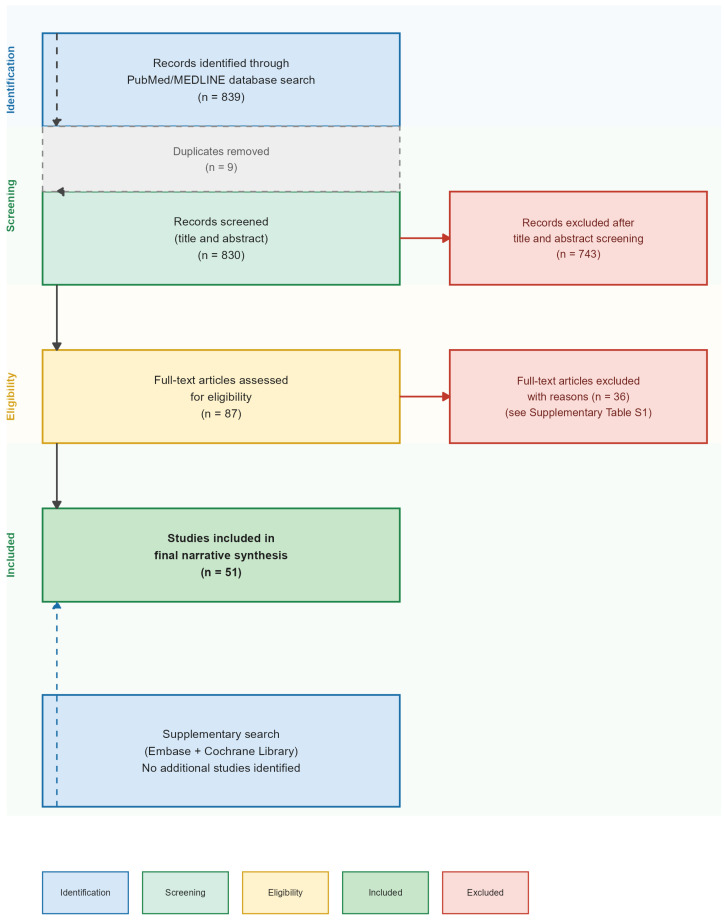
PRISMA style flow diagram of study selection. Records identified through database searching (n = 839); duplicates removed (n = 9); records screened (n = 830); records excluded after title and abstract screening (n = 743); full-text articles assessed for eligibility (n = 87); full-text articles excluded with reasons (n = 36, see Supplementary Table S1); studies included in final narrative synthesis (n = 51), n = number.

The initial search yielded 839 records. Following removal of nine duplicates, 830 records underwent title and abstract screening, of which 743 were excluded for irrelevance to the review topic. The remaining 87 articles proceeded to full-text review, and 51 studies met all eligibility criteria and were included in the final narrative synthesis. Title and abstract screening was performed by one reviewer. To address potential selection bias, a second reviewer independently screened a random 30% sample of full-text articles assessed for eligibility (26 of 87); agreement was high, and all discrepancies were resolved through discussion with a senior clinical reviewer. Reasons for exclusion of all 36 full-text articles that did not meet eligibility criteria are provided in Supplementary Table S1. The study selection process is summarised in Figure 1. A detailed summary of the 51 included studies is provided in Supplementary Table S5.

### 2.6. Data Extraction and Evidence Classification

Data were extracted using a structured approach capturing study design and setting, patient population characteristics, rehabilitation or decannulation intervention, physiological and functional outcomes, multidisciplinary care components, and health system or patient-reported outcomes where available. Included studies were classified by evidence type—randomised controlled trial, observational study, systematic review, clinical guideline, or expert consensus statement—to facilitate transparent evidence grading throughout the manuscript. Although no formal risk-of-bias tool was applied given the breadth and heterogeneity of the included evidence base, the strength and limitations of individual studies were considered during synthesis. To enhance transparency, all key interventions and clinical thresholds are classified using a simplified three-tier evidence framework: Tier 1—randomised controlled trials or multicentre prospective studies; Tier 2—observational cohort studies and clinical practice guidelines; Tier 3—single-centre studies, expert consensus, or exploratory findings. Quantitative thresholds discussed in this review are presented as evidence-informed reference points supported by varying evidence tiers rather than as definitive standards of care. A summary of the evidence strength supporting key rehabilitation and decannulation thresholds is provided in Supplementary Table S2. A summary of the evidence tier labels applied to each key intervention is provided in Supplementary Table S3. Given the predominantly observational and heterogeneous nature of the evidence base, all recommendations presented in this review should be interpreted as evidence-informed guidance rather than prescriptive standards of care.

### 2.7. Synthesis Approach

A thematic narrative synthesis was conducted. Extracted evidence was organised into six predefined clinical domains reflecting the rehabilitation-to-decannulation pathway: patient-related factors; medical management and multidisciplinary care; respiratory rehabilitation interventions; health system and resource considerations; decannulation assessment and process; and post-decannulation outcomes and quality of life. Themes were pre-specified based on clinical relevance to the rehabilitation-to-decannulation pathway and refined iteratively as evidence was reviewed. Areas of convergent evidence, clinical uncertainty, and research gaps were explicitly identified and are reported in the Limitations and Future Research Priorities sections.

## 3. Results and Discussion

### 3.1. Patient-Related and System-Level Factors Influencing Rehabilitation

The evidence synthesised across Section 3.1, Section 3.2, Section 3.3, Section 3.4, Section 3.5 and Section 3.6 derives from heterogeneous sources including randomised controlled trials, observational studies, clinical guidelines, expert consensus statements, and single-centre studies. To assist readers in interpreting the strength of individual recommendations, key interventions and thresholds are labelled throughout using the three-tier evidence classification described in Section 2.6. Readers are encouraged to prioritise recommendations supported by Tier 1 or Tier 2 evidence and to interpret Tier 3 findings as exploratory rather than practice-defining.

Throughout Section 3.1, Section 3.2, Section 3.3, Section 3.4, Section 3.5 and Section 3.6, recommendations are distinguished according to their source population. Where evidence derives directly from tracheostomized PMV patients, this is stated explicitly. Where recommendations are extrapolated from adjacent populations—including neuromuscular disease, post-stroke, acquired brain injury, or general ICU cohorts—this is clearly indicated at the point of recommendation using the notation [indirect evidence—source population stated]. Readers should apply recommendations extrapolated from adjacent populations with appropriate caution, recognising that pathophysiology, rehabilitation capacity, and decannulation trajectories may differ meaningfully across these groups.

### 3.2. Patient-Related Factors

**Figure 2:**
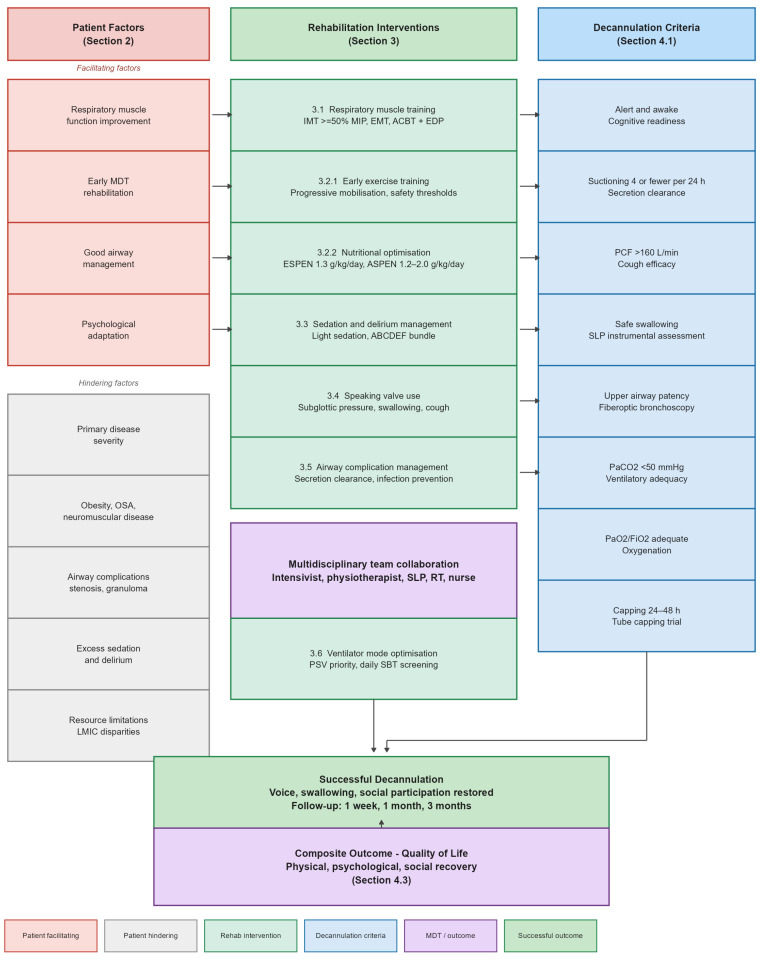
Integrated rehabilitation-to-decannulation framework for tracheostomized patients with prolonged mechanical ventilation. This figure represents a conceptual synthesis proposed by the authors based on the reviewed literature and is not a formally validated clinical framework or consensus-endorsed guideline recommendation. It is intended to illustrate the relationship between patient-related factors (Section 3.2), evidence-based rehabilitation interventions (Section 3.5), decannulation readiness criteria (Section 3.6.1), and composite patient outcomes, and should be adapted to local resources and patient characteristics. ABCDEF = ICU liberation bundle; ACBT = active cycle of breathing techniques; ASPEN = American Society for Parenteral and Enteral Nutrition; EDP = external diaphragm pacing; EMT = expiratory muscle training; ESPEN = European Society for Clinical Nutrition and Metabolism; IMT = inspiratory muscle training; LMIC = low- and middle-income countries; MDT = multidisciplinary team; MIP = maximal inspiratory pressure; OSA = obstructive sleep apnoea; PaCO_2_ = partial pressure of arterial carbon dioxide; PaO_2_/FiO_2_ = oxygenation index; PCF = peak cough flow; PSV = pressure support ventilation; QoL = quality of life; RT = respiratory therapist; SBT = spontaneous breathing trial; SLP = speech–language pathologist.

Understanding patient-specific factors that influence rehabilitation trajectory and decannulation outcomes is the essential foundation for clinical decision-making in tracheostomized PMV patients. These factors operate across three interconnected dimensions—the underlying disease and its physiological consequences, the resulting changes in respiratory mechanics and muscle function, and the patient’s psychological status and capacity for self-management. The integrated relationship between these intervention domains, patient factors, and decannulation readiness criteria is illustrated in Figure 2. This framework is a conceptual synthesis proposed by the authors based on the reviewed literature and has not undergone formal external validation or consensus endorsement.

#### 3.2.1. Primary Disease, Comorbidities, and Risk Factors for Weaning Failure

PMV accelerates systemic muscle wasting and prolongs ventilator dependence through multiple converging mechanisms—the primary disease process, systemic inflammation, prolonged immobilisation, and ventilator-induced suppression of spontaneous respiratory effort. Following tracheostomy, non-invasive ventilation (NIV) and high-flow heated humidification can assist the transition toward MV liberation. Diaphragmatic weakness and global respiratory muscle insufficiency represent the most directly modifiable barriers to decannulation. Obesity hypoventilation syndrome and obstructive sleep apnea (OSA) impose persistent mechanical loads that may persist even after the primary acute illness resolves, placing patients at sustained risk of respiratory failure [12]. Underlying chronic neuromuscular diseases—including amyotrophic lateral sclerosis, Guillain-Barré syndrome, and high cervical spinal cord injury—present a distinct challenge: respiratory rehabilitation can improve functional muscle capacity and support decannulation, but the progressive or irreversible nature of these diseases may ultimately preclude complete weaning [13].

For patients with chronic neuromuscular respiratory failure, Brown (2020) proposed a structured transitional pathway: patients achieving a peak cough flow (PCF) greater than 160 L/min may be supported toward decannulation using non-invasive positive pressure ventilation (NIPPV) [14]. The recommended approach involves daytime cuff deflation and tube capping trials with nocturnal NIV support, gradually transitioning toward complete decannulation as tolerance improves. For patients at high risk of recurrent respiratory failure, a tracheostomy button to maintain stoma patency for emergency airway access represents a clinically pragmatic intermediate step—restoring verbal communication, reducing infection risk, and improving functional independence.

#### 3.2.2. Respiratory Mechanics and Muscle Function Following Tracheostomy

Tracheostomy itself induces several favourable physiological changes compared to endotracheal intubation. The larger-bore cannula reduces dead space and airway resistance, improves expiratory flow, enhances secretion clearance, and facilitates oral and bronchopulmonary hygiene—all contributing to improved respiratory mechanics and a more favourable environment for weaning and rehabilitation [15]. Recovery of respiratory muscle function—particularly diaphragmatic function—is one of the central physiological prerequisites for decannulation. Complementary approaches targeting neuromuscular activation have been explored in tracheostomized patients. Zhang et al. (2024) evaluated a protocol combining external diaphragm pacing (EDP) with electroacupuncture at Back-Shu acupoints in post-stroke tracheostomized patients, reporting improvements in lung function and decannulation success rates [16]. These findings derive predominantly from single-centre Chinese studies with limited sample sizes and should be considered hypothesis-generating rather than standard-of-care recommendations pending larger, controlled trials.

#### 3.2.3. Psychological Status and Patient Self-Management Capacity

The psychological dimension of tracheostomy recovery is consistently underestimated in clinical practice. Tracheostomized patients report substantially higher rates of depression and anxiety compared with the general population [13,17]. The incidence of adjustment disorders is high, and psychological distress is strongly correlated with impaired quality of life. Risk factors include young age, female gender, pre-existing psychological conditions, prolonged cannula duration, voice disorders, and inadequate social support. Mc Mahon et al. (2023) highlighted that post-tracheostomy quality of life and return to social roles are significant determinants of rehabilitation effectiveness—underscoring that psychological recovery is not a secondary concern but a primary rehabilitation outcome [18]. The impact of tracheostomy on quality of life and patient-centred outcomes is discussed in detail in Section 3.4. As increasing numbers of tracheostomy-dependent patients are discharged home, structured self-management education—covering airway care, emergency management, equipment maintenance, and psychosocial adaptation—is essential for safe discharge and sustained rehabilitation engagement [13,17]. Taken together, these patient-specific factors define the clinical landscape within which the rehabilitation interventions described in Section 3.5 must be planned and individually tailored.

### 3.3. Medical Management Factors

#### 3.3.1. Timing of Tracheostomy and Its Impact on Rehabilitation

The optimal timing of tracheostomy in mechanically ventilated patients remains one of the most actively debated questions in critical care medicine, with early tracheostomy defined by most studies as occurring within 10 days of intubation. Several lines of evidence support earlier tracheostomy as beneficial for rehabilitation engagement. Smailes et al. (2022) demonstrated that early tracheostomy (MV < 10 days) significantly reduced MV duration and hospital stay, enabled earlier strength and mobility training, and improved patient satisfaction in adult ICU patients with severe burns [19]. Sutt et al. (2020) found that 88% of patients who underwent early tracheostomy were able to perform out-of-bed exercises by ICU discharge, with rehabilitation commencing a median of 6.2 days earlier—a clinically meaningful advantage in the context of PMV-associated deconditioning [20]. However, evidence on survival benefit is less clear: a meta-analysis by Han et al. (2024) concluded that early tracheostomy does not improve survival in critically ill patients expected to require prolonged MV [21]. Rossi et al. (2025) similarly found no significant reduction in ICU length of stay but confirmed that tracheostomy facilitates more effective patient management during weaning by enabling oral hygiene, improving mobility, and restoring verbal communication and oral feeding [22].

The available evidence suggests a pragmatic position: for selected patients in whom prolonged MV is anticipated, earlier tracheostomy—generally within 10 days—may expand the rehabilitation window and reduce sedation burden, although it does not improve survival [Tier 2, indirect evidence—primarily severe burns and trauma ICU cohorts]. It is important to note that the evidence supporting early tracheostomy derives predominantly from selected populations including severe burns and trauma patients; its applicability to broader PMV populations dominated by chronic respiratory failure, neuromuscular disease, or post-surgical presentations remains uncertain and should not be assumed without individualised clinical assessment. Timing decisions should always be individualised through multidisciplinary team assessment.

#### 3.3.2. Multidisciplinary Team Collaboration and Structured Care Protocols

Successful decannulation requires coordinated, structured collaboration across intensivists, rehabilitation therapists, speech–language pathologists (SLPs), respiratory therapists, and specialised nursing staff. SLPs play a particularly critical role: their assessment and management of dysphagia, voice restoration, and upper airway function directly inform the decision to proceed with cuff deflation and tube capping trials. Despite demonstrable impact, SLP services remain underutilised. Consistent improvement in tube downsizing and decannulation rates has been observed across cohorts utilising SLP services, with SLP involvement associated with faster decannulation, higher rates of safe oral diet initiation, and reduced hospital length of stay [23]. Following the establishment of an MDT SLP-led tracheostomy team in a Level 1 trauma ICU, decannulation was nearly three times more likely to occur compared to the pre-team period [24]. Healthcare professionals’ understanding of tracheostomy-related risks, complications, and care implementation is a critical determinant of patient outcomes [25,26]. Structured, protocol-driven care approaches have demonstrated tangible benefits—the American Association for Respiratory Care (AARC) has reported reductions in tracheostomy-related complications, decreased time to decannulation, increased speaking valve use, and improved patient quality of life with dedicated tracheostomy care teams [6]. The organisational model of care—tracheostomy timing and MDT collaboration—therefore directly determines the conditions under which the specific rehabilitation interventions described in Section 3.5 can be effectively delivered.

### 3.4. Healthcare System, Resource Factors, and Quality of Life Outcomes

The clinical and research focus on tracheostomized PMV patients has historically centred on physiological endpoints—ventilator liberation, infection prevention, and airway patency. Two broader contextual factors, however, exert a profound and often underappreciated influence on rehabilitation trajectories and decannulation outcomes: the healthcare system environment in which care is delivered, and the quality of life framework within which treatment success is defined. Neither is addressable through clinical intervention alone—both require systemic, institutional, and policy-level responses.

Significant disparities exist in the quality, accessibility, and outcomes of tracheostomy care across healthcare systems stratified by national income level. In high-income countries (HICs), quality improvement initiatives—most notably through the Global Tracheostomy Collaborative (GTC), established in 2012—have demonstrated measurable improvements in patient outcomes through five key drivers: multidisciplinary joint decision-making, standardised care protocols, staff education, patient and family involvement, and outcome-based metrics [27]. In contrast, across 18 published studies representing 10 low- and middle-income countries (LMICs), tracheostomy care is constrained by resource limitations including inconsistent access to running water and electricity, prohibitive financial costs of follow-up care, literacy barriers, and a near-complete absence of comparable quality improvement interventions. Rehabilitation services—including respiratory physiotherapy, SLP-led swallowing assessment, and structured decannulation protocols—that are considered standard care in high-income settings are frequently unavailable or inaccessible in LMIC environments [27]. A multi-stakeholder global survey confirmed that education and care gaps are compounded by workforce competency deficits, absence of standardised care pathways, and systemic inequities disproportionately affecting underserved settings [28]. These findings underscore that the barriers to successful decannulation in LMICs are not primarily clinical—they are structural. Addressing them requires coordinated investment in equipment provision, workforce training, and the adaptation of evidence-based protocols to resource-limited contexts.

The prevailing clinical framework—which equates treatment success with survival and successful decannulation—captures only a fraction of what matters to patients and their families. Calderone et al. (2025) demonstrated that adjustment disorders are highly prevalent among tracheostomized patients and that psychological distress is strongly correlated with impaired quality of life, regardless of whether decannulation was achieved [17]. Mc Mahon et al. (2023) established that post-tracheostomy quality of life and return to social roles are significant determinants of rehabilitation effectiveness—meaning that a patient who achieves decannulation but remains socially isolated and psychologically distressed has not achieved a fully successful outcome [18]. Outcome measures for tracheostomized PMV patients should extend beyond physiological parameters to encompass patient-reported outcomes (PROMs) including communication capacity, swallowing function, psychological well-being, social participation, and return to meaningful roles. Incorporating validated PROMs into clinical protocols and research frameworks for this population represents both a clinical and scientific priority. True treatment success requires that patients achieve meaningful physical, psychological, and social recovery alongside biological endpoints. Understanding these system-level and patient-centred dimensions of tracheostomy rehabilitation provides the essential context for evaluating the evidence-based interventions synthesised in the following section.

### 3.5. Respiratory Rehabilitation Interventions

#### 3.5.1. Respiratory Muscle Training

Prolonged mechanical ventilation predisposes patients to progressive respiratory muscle dysfunction. The diaphragm demonstrates detectable ultrasound-confirmed atrophy as early as 18 to 69 h after MV initiation, driven by disuse atrophy, oxidative stress, and proteolysis [29]. Diaphragmatic dysfunction is independently associated with prolonged weaning time and failure to liberate from MV [29]. In a prospective study of 124 patients ventilated for more than 24 h, Bissett et al. (2019) demonstrated that 54% had clinically significant inspiratory muscle weakness prior to weaning, independently associated with one-year mortality [3].

#### 3.5.2. Inspiratory Muscle Training

Targeted inspiratory muscle training (IMT) is currently supported by the strongest available evidence among the interventions reviewed for respiratory muscle rehabilitation in mechanically ventilated patients (Tier 1–2). Multidisciplinary practice guidelines recommend threshold loading as the preferred IMT modality, with training intensity prescribed as a proportion of maximal inspiratory pressure (MIP), measurable via the tracheostomy or endotracheal tube using a ventilator or handheld manometer [10]. The protocol with the highest level of evidence consists of high-intensity threshold loading: six breaths per set, five sets per session, at a load of no less than 50% of MIP, performed daily with progressive load increases under therapist supervision [10,30]. As MIP improves, loads must be systematically adjusted to sustain the training stimulus. IMT-driven improvement in inspiratory muscle strength may support decannulation readiness by enhancing the patient’s capacity to sustain spontaneous breathing, generate effective cough, and tolerate cuff deflation and tube capping trials. It is important to note that even patients managed primarily on spontaneous ventilation modes retain significant risk of inspiratory muscle weakness—Bissett et al. (2019) demonstrated that one-third of such patients still had clinically significant weakness—underscoring that ventilator mode selection alone is insufficient and must be combined with active IMT [3]. Beyond threshold loading, Li et al. (2025) [31] evaluated a protocol combining the Active Cycle of Breathing Techniques (ACBT) with External Diaphragm Pacing (EDP) in tracheostomized stroke patients, demonstrating improvements in diaphragmatic excursion, diaphragmatic thickening fraction (DTF), and PaO_2_. Evidence quality is currently limited to single-centre observational data [31].

#### 3.5.3. Expiratory Muscle Training

While IMT has received considerable research attention, expiratory muscle training (EMT) remains comparatively understudied (Tier 3—insufficient evidence) despite its direct clinical consequences for decannulation. Effective cough—which depends on expiratory muscle strength—is a prerequisite for safe decannulation: PCF greater than 160 L/min is one of the established decannulation readiness criteria. Patients with adequate inspiratory weaning parameters who nonetheless fail decannulation due to inability to clear secretions represent an important and underappreciated clinical failure mode. Current evidence on EMT protocols in tracheostomized PMV patients remains insufficient, and the optimal training method, intensity, and timing relative to IMT has not been systematically established.

#### 3.5.4. Complementary Approaches

Electroacupuncture has been explored as a complementary modality for diaphragmatic rehabilitation. Zhu et al. (2020) reported improvements in diaphragmatic function following electroacupuncture in post-stroke tracheostomized patients [32]. Available evidence derives predominantly from single-centre studies conducted almost exclusively in post-stroke patients within Chinese clinical settings, with small sample sizes and limited methodological rigor. Their applicability to tracheostomized PMV patients with diverse underlying conditions—including COPD, cardiac failure, and post-surgical presentations—is highly limited. Electroacupuncture is not an internationally endorsed standard of care for tracheostomized or PMV patients (Tier 3, exploratory). These findings should be interpreted as culture-specific, hypothesis-generating evidence and are included here solely to provide a comprehensive overview of all interventions studied in this area. They are not recommended for routine clinical application outside the specific populations and settings in which they have been studied.

#### 3.5.5. Physical Rehabilitation and Nutritional Optimisation

##### Early Exercise Training

Tracheostomized patients with PMV experience a compounding cycle of muscle deconditioning: the primary disease, prolonged immobilisation, mechanical ventilation, and systemic inflammation each independently contribute to muscle wasting, diaphragmatic dysfunction, and physical deconditioning. Early exercise rehabilitation, initiated as soon as clinical condition permits, is now recognised as a core strategy for interrupting this cycle and creating the physiological conditions necessary for successful weaning and decannulation (Tier 2). The foundational principle is progressive mobilisation structured according to physiological capacity, proceeding from passive limb exercises and positioning, through active-assisted exercises, sitting at the bedside, standing with support, and, where feasible, ambulation. Before initiating active mobilisation, minimum safety thresholds should be confirmed: haemodynamic stability (heart rate 40–130 bpm, mean arterial pressure ≥ 65 mmHg), adequate oxygenation (SpO_2_ ≥ 88% on current ventilator settings), absence of agitation, and secure airway access. Dong et al. (2021) demonstrated that early structured rehabilitation through a 6-level graded programme effectively attenuated diaphragmatic dysfunction associated with prolonged MV, reduced ICU length of stay, and improved quality of life [33]. Improved limb and trunk muscle strength supports respiratory mechanics, and improved functional capacity supports the cuff deflation and tube capping trials required for decannulation assessment.

##### Nutritional Support and Metabolic Optimisation

Nutritional status is not a background variable in the rehabilitation of tracheostomized PMV patients—it is a direct physiological determinant of respiratory muscle recovery, weaning capacity, and ultimately decannulation readiness. Critically ill patients can lose up to 20% of total muscle mass within 10 days of ICU admission—a rate of depletion that directly undermines respiratory muscle function, cough efficacy, and the physiological prerequisites for decannulation [34]. Despite the severity and clinical consequences of this depletion, total energy and protein delivery in mechanically ventilated patients consistently falls short of recommended targets in clinical practice.

The major international guidelines provide a consistent framework for nutritional management. The ESPEN 2023 revised guideline recommends progressive protein delivery to a target of 1.3 g/kg/day for critically ill patients, with higher targets in specific subgroups including elderly, obese, trauma, and acute kidney injury patients [34]. The ASPEN critical care nutrition guidelines recommend protein delivery between 1.2 and 2.0 g/kg/day, with caloric targets of 12–25 kcal/kg/day over the first 7–10 days of ICU admission [35]. Neither guideline currently provides specific nutritional recommendations tailored to the tracheostomized PMV sub-population. These targets are derived from guidelines for general critically ill patients and have not been prospectively validated in tracheostomized PMV patients as a distinct clinical entity (Tier 2, indirect evidence). Their application in this population should be considered evidence-informed rather than population-specific, and clinical teams should individualise targets based on metabolic monitoring and patient tolerance.

Both guidelines recommend indirect calorimetry as the preferred method for determining individual energy expenditure; where unavailable, VCO_2_ derived from the ventilator provides a more accurate estimate than weight-based equations alone. For tracheostomized PMV patients, enteral nutrition (EN) is the preferred route of delivery, initiated within 48 h of ICU admission where clinically feasible. Patients with high aspiration risk may benefit from postpyloric feeding. Clinicians should be vigilant for refeeding syndrome, characterised by potentially life-threatening electrolyte shifts including hypophosphataemia, hypokalaemia, and hypomagnesaemia—requiring careful monitoring and gradual escalation of nutritional delivery. Adequate protein delivery supports respiratory muscle anabolism, directly counteracting the diaphragmatic and accessory muscle atrophy that impairs SBT tolerance and capping trial success, suggesting that nutritional optimisation may contribute to decannulation readiness.

#### 3.5.6. Sedation and Delirium Management

The depth of sedation and the prevention of delirium are not peripheral concerns in the rehabilitation of tracheostomized PMV patients—they are central determinants of whether a patient can actively participate in the rehabilitation interventions described in this review. A patient who is over-sedated cannot participate in IMT, cannot engage in progressive mobilisation, cannot tolerate cuff deflation trials, and cannot demonstrate the consciousness and secretion management capacity required for decannulation assessment. Optimising sedation strategy and preventing delirium are therefore important contributors to the entire rehabilitation pathway, not adjuncts to it.

The paradigm of sedation management has shifted fundamentally over the past two decades, from deep sedation as a default to targeted light sedation as the goal-directed standard. Protocols targeting minimal sedation, maintaining patients in an awake, cooperative, and comfortable state, have been confirmed across multiple studies to shorten MV duration, reduce delirium incidence, and improve short-term prognosis [36]. For tracheostomized PMV patients specifically, light sedation is directly enabling: it permits active participation in breathing exercises, reduces ventilator dependence, facilitates communication through speaking valve use, and creates the conditions for progressive cuff deflation and tube capping trials that form the decannulation assessment pathway. In patients with ARDS, who may require low tidal volume ventilation, high positive end-expiratory pressure (PEEP), prone positioning, and occasionally neuromuscular blockade, achieving light sedation while maintaining ventilator synchrony and patient comfort demands careful individualisation. Evidence indicates that optimising ventilator parameters to reduce patient–ventilator asynchrony is more effective than deepening sedation in ARDS patients, a distinction with direct implications for rehabilitation engagement in this subgroup [36].

Delirium in the ICU is not merely a complication of critical illness—it is an active barrier to rehabilitation and decannulation. Delirium prevents patients from following instructions during IMT and mobilisation, impairs their ability to manage secretions and protect the airway, and makes cuff deflation and tube capping trials unsafe. The current evidence-based standard for ICU liberation is the ABCDEF bundle: Assess, prevent, and manage pain; Both spontaneous awakening trials and spontaneous breathing trials; Choice of analgesia and sedation; Delirium assessment, prevention, and management; Early mobility and exercise; and Family engagement and empowerment (Tier 1–2) [37]. Implementation of the ABCDEF bundle has been associated with significant reductions in delirium prevalence, shorter MV duration, reduced ICU length of stay, and improved functional outcomes—outcomes that directly support decannulation readiness. Comprehensive delirium management requires multimodal, predominantly non-pharmacological intervention. Mart et al. (2021) emphasised that effective delirium management requires coordinated strategies including early mobilisation, sleep-wake cycle promotion, cognitive stimulation, and active family involvement [38]. For tracheostomized PMV patients, these strategies are particularly important—and particularly achievable—because tracheostomy itself, by reducing sedation requirements compared to translaryngeal intubation, creates a more favourable neurological environment for delirium prevention and cognitive engagement. Pharmacological delirium management remains a secondary strategy—antipsychotic agents have not been shown to reduce delirium duration or improve outcomes in large ICU trials and should not be used as first-line prevention. Pain management is an inseparable component of delirium prevention—inadequately managed pain is a leading precipitant of agitation and delirium, and the analgesia-first approach—prioritising pain management before titrating sedation—is the current recommended paradigm [36].

#### 3.5.7. Speaking Valve Use

Tracheostomy fundamentally disrupts the physiological functions of the upper airway—voice, swallowing, and airway protection—that are central to a patient’s quality of life, dignity, and active participation in rehabilitation. Restoring these functions should be considered a primary rehabilitation goal. According to the UK Faculty of Intensive Care Medicine guidance on tracheostomy care, vocalisation should be a daily goal of care for tracheostomized patients, and speech and language therapy assessment should occur at the point when sedation is held and ventilation is being weaned [39]. The one-way speaking valve is the primary clinical tool for achieving this goal, and its benefits extend well beyond communication to directly support the physiological prerequisites for decannulation.

##### Physiological Rationale

The speaking valve functions by redirecting expiratory airflow through the upper airway rather than out through the tracheostomy—restoring subglottic pressure and laryngopharyngeal airflow to a near-physiological state. This produces multiple clinically important effects: restored subglottic positive end-expiratory pressure prevents early alveolar closure and improves ventilation-oxygenation; restored laryngopharyngeal airflow triggers the laryngeal adductor reflex, promotes effective glottic closure, and reduces aspiration risk directly relevant to the swallowing function assessment required for decannulation; and enhanced laryngeal sensory stimulation improves swallowing awareness, taste, and smell (Tier 2, indirect evidence). Han et al. (2022) provided direct biomechanical evidence that speaking valve use significantly increases the peak and duration of subglottic pressure during swallowing, increases laryngeal elevation, and accelerates airway closure—findings translating directly into reduced aspiration risk and improved swallowing safety during decannulation assessment [40,41]. It should be noted that the majority of biomechanical and swallowing-related evidence for speaking valve use derives from post-stroke and acquired brain injury cohorts (Tier 2, indirect evidence). The applicability of these findings to tracheostomized PMV patients with COPD, cardiac failure, or post-surgical presentations requires cautious interpretation. The pathway from speaking valve use to decannulation is explicit: restored subglottic pressure → improved swallowing coordination → successful swallowing assessment → cuff deflation tolerance → tube capping feasibility → decannulation readiness. Speaking valve use is not a quality of life intervention layered on top of the decannulation process—it is an integral component of it.

##### Timing, Technique, and ACV

The optimal timing of speaking valve introduction in PMV patients remains an area of active investigation. Martin et al. (2021) conducted the only RCT examining accelerated versus standard speaking valve placement following percutaneous tracheostomy, demonstrating the feasibility of earlier introduction—though the study was designed primarily to assess feasibility rather than definitive clinical outcomes, and larger confirmatory trials are needed [42]. The prerequisite for standard speaking valve use is successful cuff deflation. Before attempting cuff deflation, clinicians must rule out upper airway abnormalities—including glottic or subglottic oedema, vocal cord paralysis, severe tracheal stenosis, or post-intubation laryngeal cartilage injury—that would prevent adequate supraglottic airflow and make valve use unsafe [43]. For patients who cannot tolerate cuff deflation—due to high ventilatory requirements, haemodynamic instability, or upper airway pathology—Above Cuff Vocalisation (ACV) provides an alternative pathway to voice restoration. ACV involves delivery of exogenous airflow through the subglottic suction port to vibrate the vocal cords and produce sound without requiring cuff deflation [43]. The evidence base for ACV highlights both its potential benefits and the significant variability in clinical application and the limited, low-quality evidence currently available [44]. ACV should be considered as a clinical option in ventilator-dependent patients who would otherwise be denied the benefits of laryngopharyngeal airflow restoration during the weaning phase.

#### 3.5.8. Airway Complication Management

Airway complication management is not a passive safety function in the rehabilitation of tracheostomized PMV patients—it is a proactive, clinically active component of the decannulation pathway (Tier 2–3). Complications of tracheostomy—both early and late—can directly prevent decannulation by creating mechanical, infectious, or functional barriers in the airway. Early complications include stomal wound infection, surgical emphysema, pneumothorax, haemorrhage, and accidental decannulation. Late complications—particularly relevant to the PMV population given prolonged cannulation time—include granulation tissue formation, tracheal stenosis, tracheomalacia, vocal cord dysfunction, and tracheostomal stenosis. Each late complication can directly obstruct the decannulation pathway by preventing adequate supraglottic airflow during capping trials, causing respiratory distress during cuff deflation, or precipitating decannulation failure requiring recannulation. Clara SL et al. (2025) demonstrated in critically ill COVID-19 survivors that the incidence of laryngotracheal pathologies—including granulation tissue formation, vocal cord dysfunction, and tracheal stenosis—at one-year follow-up was clinically significant and should not be underestimated [45]. This finding has direct relevance beyond the COVID-19 population—any patient who has experienced prolonged tracheostomy cannulation is at risk for these late sequelae. Xie et al. (2025) identified pneumonia, atelectasis, and airway hyperreactivity as independent risk factors directly hindering decannulation—reinforcing the principle that the primary goal of tracheostomy airway management is to create and preserve the conditions under which decannulation becomes achievable [46].

Systematic pre-tracheostomy assessment is the most effective strategy for reducing long-term airway complication burden. High-risk factors—including obesity, limited neck mobility, history of airway surgery, history of cervical radiotherapy, known tracheal abnormality, and coagulopathy—should be identified and documented before tracheostomy is performed. Surgical tracheostomy should be favoured over percutaneous dilatational tracheostomy (PDT) in complex anatomical situations, as direct visualisation provides greater safety. PDT is generally associated with fewer wound complications and lower infection rates in standard anatomy patients, while surgical tracheostomy allows better control of tube placement and stoma formation, which may reduce long-term stomal stenosis and granulation tissue risk in patients anticipated to require prolonged cannulation. The implications of this choice for rehabilitation trajectory and decannulation outcomes have not been systematically studied and represent an important evidence gap.

High-quality active airway management throughout rehabilitation requires three critical priorities: (1) Secretion management: effective airway clearance requires adequate systemic hydration, optimised airway humidification, regular suctioning using aseptic technique, and adjunctive strategies including manual chest physiotherapy, assisted cough techniques, and in selected patients mechanical insufflation–exsufflation devices. Peak cough flow is directly dependent on secretion clearance capacity—improving secretion management is therefore a direct investment in decannulation readiness. (2) Infection prevention: ventilator-associated pneumonia (VAP) and tracheostomy-related infections are independent barriers to decannulation by increasing secretion burden, causing systemic deterioration, and delaying rehabilitation engagement. Bundle-based infection prevention strategies—including head-of-bed elevation, oral hygiene protocols, subglottic secretion drainage, and minimal sedation—should be applied rigorously throughout the tracheostomy period. (3) Surveillance for late complications: clinicians should maintain a low threshold for fiberoptic bronchoscopic assessment when decannulation fails unexpectedly or when patients develop progressive respiratory distress, stridor, or unexplained failure to progress with capping trials.

#### 3.5.9. Ventilator Mode Optimisation and Weaning

Ventilator mode selection and weaning strategy are among the most consequential clinical decisions in the management of tracheostomized PMV patients. The fundamental physiological principle governing ventilator mode selection is as follows: diaphragmatic inactivity drives atrophy, and atrophy drives ventilator dependence. Controlled mechanical ventilation (CMV), by rendering the diaphragm completely inactive, induces rapid-onset ventilator-induced diaphragmatic dysfunction (VIDD) through oxidative stress, protease activation, and accelerated myofibrillar proteolysis. Detectable diaphragmatic atrophy has been documented after as few as 18 h of controlled ventilation in humans, and diaphragmatic atrophy of more than 10% has been directly associated with longer duration of controlled ventilation and higher PEEP levels [47,48]. Pressure support ventilation (PSV), by contrast, preserves diaphragmatic contractile activity—allowing patient-triggered breaths that maintain neuromuscular activation and attenuate the proteolytic cascade (Tier 2). PSV does not eliminate VIDD entirely—high-level PSV with excessive over-assistance can still produce disuse atrophy—but the magnitude and rate of atrophy are substantially lower than with CMV [3]. For tracheostomized PMV patients, spontaneous breathing modes—primarily PSV—should be prioritised as soon as the patient’s clinical condition permits, and the level of pressure support should be titrated to maintain adequate diaphragmatic effort without either over-assistance (atrophy risk) or under-assistance (fatigue risk).

The spontaneous breathing trial (SBT) is the clinical cornerstone for identifying patients ready to be liberated from mechanical ventilation. Burns et al. (2024) demonstrated in a large RCT that daily screening combined with standardised SBT protocols significantly accelerates the weaning process (Tier 1) [49]. Pressure support SBT is generally better tolerated than T-piece SBT in tracheostomized PMV patients. Liu et al. (2022) further demonstrated that SBT reduces unnecessary prolonged ventilation and decreases failed extubation and decannulation attempts [50]. Critically, successful SBT is a necessary but insufficient condition for decannulation—it confirms ventilator liberation readiness, not decannulation readiness. Swallowing function, secretion clearance, cough efficacy, and upper airway patency must all be assessed independently. The sequential pathway from ventilator dependence to decannulation proceeds: CMV → PSV → SBT tolerance → ventilator liberation → cuff deflation trials → tube capping trials → decannulation readiness assessment → decannulation. Multidisciplinary daily screening to assess readiness for progression at each step is the most evidence-supported approach to accelerating this pathway while maintaining patient safety [49]. This sequential rehabilitation-to-decannulation pathway is synthesised visually in Figure 2.

### 3.6. Decannulation—Assessment, Process, and Post-Procedural Outcomes

#### 3.6.1. Decannulation Readiness Assessment

Decannulation represents far more than the removal of a tracheostomy tube—it is a transformative clinical milestone that marks the restoration of physiological airway independence, voice, swallowing, and the capacity for meaningful social participation. Research among adult tracheostomized ICU patients has demonstrated that voice loss profoundly affects patients’ ability to communicate their care and comfort needs, generates intense psychological distress, and fundamentally undermines their sense of recovery and participation in their own care. Conversely, restoration of voice—which decannulation enables—is directly associated with patient-reported improvements in mood, outlook, self-esteem, and quality of life [51]. Decannulation therefore represents restoration of verbal communication, safe swallowing, and capacity for social participation—outcomes that extend beyond physiological endpoints.

Despite its clinical importance, decannulation remains one of the least standardised procedures in critical care. Unified evidence-based guidelines for decannulation are currently lacking [6], and clinical practice varies considerably across institutions and countries. A multicentre observational study from Italian intensive care settings reported that only 22% of tracheostomized patients achieved decannulation despite having achieved ventilator liberation, and a further 26% were discharged from hospital with the tracheostomy cannula still in place [52]. This figure should be interpreted as context-specific rather than globally representative—specialised weaning and rehabilitation centres report substantially higher decannulation rates, typically between 40% and 70%, depending on patient selection, multidisciplinary team structure, and institutional protocols. The wide variability across settings suggests that system-level factors—including the availability of structured decannulation protocols, multidisciplinary team composition, and rehabilitation resources—play an important role alongside patient physiology in determining decannulation outcomes.

#### 3.6.2. Redefining Decannulation Success

The prevailing clinical definition of decannulation success—absence of recannulation within hours or days of tube removal—is insufficient. Winiker et al. (2026) argue compellingly that decannulation success should be conceptualised as a composite, multidimensional outcome encompassing physiological, psychological, and temporal dimensions [53]. A comprehensive definition must include surveillance for complications occurring within three months post-decannulation—altered respiratory rate, respiratory insufficiency, respiratory infection, new-onset hypertension, new neurological symptoms, new sleep-disordered breathing, intratracheal complications such as granulation tissue, stomal complications—and their consequences including reintubation, recannulation, and death. This expanded definition has direct implications for how post-decannulation follow-up is structured, as addressed in Section 3.6.6.

#### 3.6.3. Clinical Assessment Criteria for Decannulation Readiness

Based on current evidence and expert consensus, decannulation readiness assessment should systematically evaluate five domains, interpreted by a multidisciplinary team in the context of each patient’s individual clinical trajectory.

(1) Neurological and cognitive readiness—the patient should be alert and awake with sufficient cognitive function to follow simple instructions, manage oral secretions voluntarily, and cooperate with cuff deflation and capping trials (Tier 2–3, expert consensus and observational data). Patients who cannot protect their airway or respond to respiratory distress are generally considered unsuitable for decannulation regardless of respiratory parameters.
(2) Secretion management capacity—a suctioning frequency of four or fewer times in a 24-h period is commonly used in clinical protocols as an indicator of adequate spontaneous secretion clearance capacity
(3) Tier 3, expert consensus; Devaraja et al., 2024 [54]). This threshold derives from observational data in general tracheostomy cohorts and has not been validated as an optimal cutoff in heterogeneous PMV populations; it should be interpreted as a clinical reference point rather than an absolute standard.
(4) Cough efficacy—a PCF greater than 160 L/min is a commonly used evidence-informed threshold for adequate cough strength to clear secretions and protect the airway post-decannulation (Tier 2–3, indirect evidence; Ge et al., 2024 [55]). This threshold is supported primarily by observational studies in spontaneously breathing tracheostomized adults and has not been prospectively validated as a universal criterion in heterogeneous tracheostomized PMV cohorts. PCF can be measured directly through the tracheostomy tube using a peak flow meter.
(5) Swallowing function—the patient should demonstrate safe swallowing of liquids or semi-solid foods without aspiration before decannulation. Swallowing assessment should be conducted by an SLP using instrumental evaluation—laryngoscopy and videofluoroscopy—rather than bedside clinical assessment alone, which has insufficient sensitivity for silent aspiration detection (Tier 2, indirect evidence; Gallice et al., 2024 [4]). The majority of evidence supporting instrumental swallowing assessment derives from post-stroke and acquired brain injury populations; its applicability to broader PMV cohorts requires cautious interpretation.
(6) Upper airway patency and gas exchange—fiberoptic bronchoscopy may be used to exclude structural upper airway pathology including granulation tissue, tracheal stenosis, subglottic oedema, and tracheomalacia [Tier 3, expert consensus and case series]. Its independent predictive value for decannulation outcomes and optimal timing remain uncertain; clinical decisions should be individualised based on patient circumstances. A PaCO_2_ below 50 mmHg on room air or low-flow supplemental oxygen is commonly used as a reference indicator of sufficient ventilatory drive and capacity (Tier 2–3, indirect evidence from general ventilator weaning literature); this threshold has not been validated specifically in tracheostomized PMV patients and should be interpreted accordingly.

**Figure 3:**
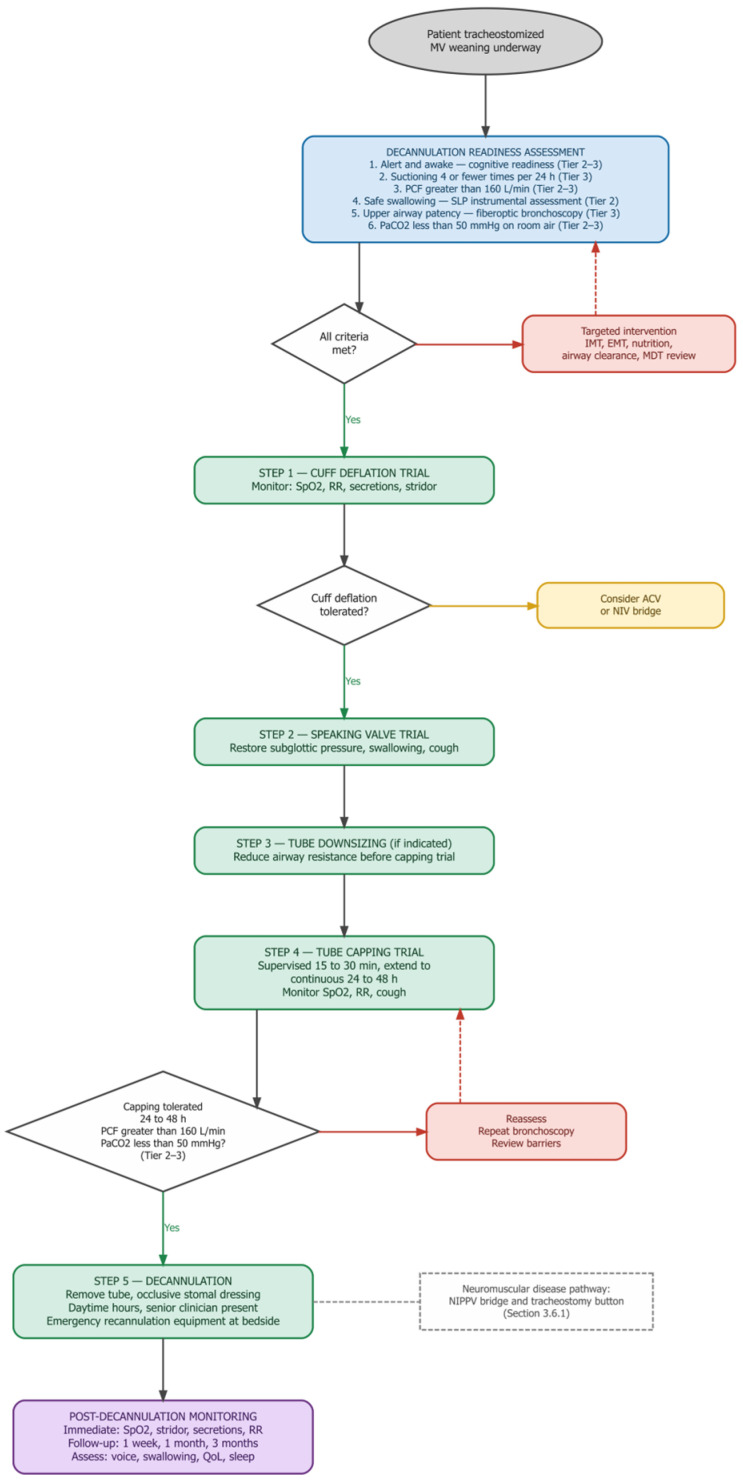
Decannulation decision algorithm for tracheostomized patients with prolonged mechanical ventilation. This figure represents a conceptual framework synthesised by the authors based on the reviewed literature and is intended as an evidence-informed clinical aid rather than a formally validated algorithm or guideline recommendation; institutions should adapt it to their own resources and clinical context. Tier labels indicate evidence strength per the classification in Section 2.6 (Tier 1 = RCT/multicentre prospective; Tier 2 = observational/guidelines; Tier 3 = single-centre/expert consensus/exploratory). ACV = above cuff vocalisation; EMT = expiratory muscle training; IMT = inspiratory muscle training; MDT = multidisciplinary team; NIV = non-invasive ventilation; NIPPV = non-invasive positive pressure ventilation; PCF = peak cough flow; QoL = quality of life; RR = respiratory rate; SLP = speech–language pathologist; SpO2 = peripheral oxygen saturation.

The five assessment domains and their clinical thresholds are summarised in Figure 3.

#### 3.6.4. Multidisciplinary Assessment Framework

No single clinician or discipline possesses the full range of expertise required to conduct a comprehensive decannulation readiness assessment. The framework should incorporate the coordinated input of intensivists or pulmonologists confirming ventilatory and gas exchange parameters, respiratory therapists assessing cough flow and secretion management capacity, SLPs conducting instrumental swallowing assessment, physiotherapists confirming functional capacity and mobilisation status, and nursing staff providing longitudinal observation of secretion frequency and patient behaviour. The critical role of multidisciplinary team collaboration in supporting this process is discussed in Section 3.3.2. This multidisciplinary assessment should be conducted according to a structured, institution-level protocol—not on an ad hoc basis—to ensure consistency and reduce the variation that currently characterises decannulation practice internationally [6,56]. A structured decannulation decision algorithm incorporating these assessment steps is presented in Figure 3.

#### 3.6.5. The Decannulation Process

Once decannulation readiness criteria have been met as described in Section 3.6.1, the decannulation process proceeds through a structured, stepwise sequence as shown in Figure 3. This algorithm represents a conceptual synthesis of the available literature and should be interpreted as an evidence-informed clinical aid rather than a formally validated or consensus-endorsed pathway. Each step is both a clinical assessment and a therapeutic intervention—confirming the patient’s capacity for the next level of airway independence while building the physiological tolerance required for complete tube removal.

Step 1—Cuff deflation trial: the first step is transitioning from a sealed, cuffed airway to one permitting airflow through the upper airway alongside the tracheostomy tube. Contraindications include significant upper airway obstruction, uncontrolled aspiration, requirement for high positive pressure ventilatory support incompatible with cuff deflation, and severe haemodynamic instability. Monitor for intolerance—respiratory distress, oxygen desaturation below 90%, stridor, or inability to manage secretions. Tolerated periods should be progressively extended.

Step 2—Speaking valve trial and tube downsizing: once cuff deflation is tolerated, speaking valve placement should be prioritised—both for voice restoration and its physiological role in preparing the airway for tube capping. Speaking valve tolerance serves as a functional assessment of upper airway patency and expiratory flow capacity [6]. Where larger-diameter tubes are in situ, downsizing before capping reduces airway resistance and should be performed over a guidewire by an experienced clinician.

Step 3—Tube capping trial: tube capping is the definitive test of capacity for complete airway independence. Capping trials should begin with supervised periods of 15 to 30 min, advancing to continuous capping for a minimum of 24 to 48 h [4,54]. The patient should breathe comfortably through the upper airway, maintain oxygen saturation without supplemental oxygen, cough effectively, and tolerate sleep through at least one capping period. Failure of capping trials should trigger systematic reassessment including repeat fiberoptic bronchoscopy to exclude newly developed airway pathology.

Step 4—Decannulation: once the patient has tolerated continuous tube capping for 24 to 48 h with PCF > 160 L/min and confirmed PaCO_2_ below 50 mmHg, decannulation is recommended during daytime hours when the full clinical team is available, with resuscitation equipment and emergency recannulation materials immediately available for the first 24 h [54,55]. The tube is removed, the stoma dressed with an occlusive dressing, and the patient instructed to apply manual pressure during coughing and phonation until stomal closure.

Brown (2020) proposed a modified pathway for patients with chronic neuromuscular respiratory failure—patients achieving PCF greater than 160 L/min may be supported toward decannulation using NIPPV—daytime cuff deflation and capping trials with nocturnal NIV support [14]. For patients at high risk of recurrent respiratory failure, a tracheostomy button maintains stoma patency while restoring the benefits of decannulation.

#### 3.6.6. Post-Decannulation Monitoring and Outcomes

Decannulation is not the end of the clinical journey—it is the beginning of a new phase carrying its own risks, monitoring requirements, and rehabilitation goals. The first 24 to 48 h following tube removal represent the highest-risk period for decannulation failure. Monitoring should include continuous pulse oximetry, regular respiratory rate assessment, observation for stridor or increased work of breathing, and evaluation of cough efficacy and secretion management. Clinical thresholds triggering urgent reassessment and consideration of recannulation include: oxygen saturation below 90% on low-flow supplemental oxygen, audible stridor at rest, inability to manage secretions with spontaneous cough, PaCO_2_ rising above 50 mmHg, or signs of significant respiratory distress. Resuscitation equipment and emergency recannulation materials must remain at the bedside for the first 24 h. Stoma patency persists for several hours after tube removal, providing a window for emergency recannulation without surgical intervention if required.

Structured follow-up at one week, one month, and three months post-decannulation should assess respiratory function, stomal healing, voice quality, swallowing safety, and sleep-disordered breathing, which may unmask or exacerbate previously subclinical obstructive sleep apnea following restoration of upper airway anatomy. Winiker et al. (2026) define successful decannulation as a composite outcome requiring a minimum three-month surveillance window, encompassing altered respiratory rate, respiratory insufficiency, respiratory infection, new neurological symptoms, new sleep-disordered breathing, intratracheal complications, and stomal complications [53]. Long-term mortality data confirm that risk extends well beyond hospital discharge [54].

Psychological outcomes—including adjustment disorders, depression, and anxiety—remain prevalent months following decannulation and require active clinical attention integrated into the post-decannulation pathway, as discussed in Section 3.2.3. The ultimate measure of decannulation success extends beyond physiological parameters to encompass voice restoration, psychological well-being, and social reintegration, as outlined in Section 3.6.1. Patient-reported outcome measurement covering communication, psychological well-being, and social participation must be incorporated into follow-up protocols alongside physiological parameters. Recannulation should be decided on clinical grounds—respiratory decompensation, inability to protect the airway, or failure to manage secretions—and should not be delayed to preserve the decannulation outcome. Recannulation is a safety decision, not a clinical failure; patients and families should be counselled that the pathway may not be linear and that recannulation does not preclude subsequent successful decannulation.

## 4. Limitations of This Review

This review has several methodological and evidentiary limitations that should be considered when interpreting its findings and applying its recommendations to clinical practice. The literature search was restricted to a single database—PubMed/MEDLINE—which may have resulted in omission of relevant studies indexed exclusively in Embase, Cochrane Library, Web of Science, or Scopus. A targeted supplementary search of Embase and the Cochrane Library was performed, but the possibility of omitted studies cannot be completely excluded. PubMed does not comprehensively index speech–language pathology and nursing literature, which are better represented in CINAHL and ASHA databases. The restriction to English and Chinese language publications may have introduced language bias. As a narrative review, no formal quality appraisal tool—such as the Cochrane Risk of Bias tool, Newcastle–Ottawa Scale, or GRADE framework—was applied to included studies. The strength of evidence underlying individual recommendations therefore varies considerably across sections, and recommendations based on observational data, expert consensus, or single-centre studies should be distinguished from those supported by higher-quality evidence. Study selection was performed by one reviewer, with a second reviewer independently screening a random 30% sample of full-text articles to assess selection consistency; all discrepancies were resolved through discussion with a senior clinical reviewer. Although this process reduces selection bias, the inclusion of grey literature and expert consensus documents—where subjective eligibility judgments are inherently more variable—remains a methodological consideration that readers should bear in mind when interpreting the findings.

A pervasive limitation of the existing evidence base—not of this review specifically, but of the field as a whole—is the substantial heterogeneity of study populations. Many recommendations are extrapolated from populations adjacent to but not identical to tracheostomized PMV patients. Nutritional recommendations derive primarily from general critically ill patients and specific disease subgroups—ALS and major trauma; speaking valve evidence derives predominantly from post-stroke and acquired brain injury populations; and TCM-based interventions have been studied almost exclusively in post-stroke patients in single-centre Chinese settings, limiting international applicability. No specific nutritional protocols, ventilator weaning algorithms, or decannulation criteria have been prospectively validated in the tracheostomized PMV population as a distinct clinical entity. The evidence base derives predominantly from high-income country settings—substantially limiting applicability to LMIC environments where most of the world’s tracheostomized PMV patients reside. The absence of validated PROMs specifically designed for tracheostomized PMV patients limits the field’s ability to evaluate whether interventions that succeed on physiological parameters also translate into outcomes that matter to patients. Supplementary Table S4 summarises which key recommendations in this review are derived directly from tracheostomized PMV populations and which are extrapolated from adjacent populations.

The clinical algorithms and conceptual frameworks presented in Figure 2 and Figure 3 have not undergone external prospective validation or formal Delphi consensus. They should be regarded as evidence-informed tools to support clinical reasoning rather than prescriptive clinical pathways, and future research should attempt prospective evaluation of these frameworks.

## 5. Future Research Priorities

Five specific, actionable research priorities emerge from the limitations identified above.

(1) Dedicated RCTs in the tracheostomized PMV population: the most urgent research need is the conduct of rigorously designed randomised controlled trials in homogeneous tracheostomized PMV patient subgroups. Priority trial questions include the optimal IMT protocol intensity and frequency, the benefit of combined IMT and EMT versus IMT alone, the effect of nutrition-as-therapy protocols on decannulation rates, and the comparative effectiveness of different weaning strategies in tracheostomized versus intubated patients.
(2) Expiratory muscle training research: EMT is critically underinvestigated relative to its clinical importance. PCF—which depends on expiratory muscle strength—is a primary decannulation criterion, yet no dedicated RCTs have established the optimal EMT protocol, training intensity, or timing relative to IMT in tracheostomized PMV patients. Given the direct mechanistic link between expiratory muscle strength, cough efficacy, and the PCF > 160 L/min decannulation threshold, EMT research should be considered a priority investment for the field.
(3) Prospective validation of decannulation readiness criteria: a multicentre prospective study establishing the predictive validity of specific decannulation criteria—including the PCF threshold, suctioning frequency, and capping trial duration—in a diverse PMV population would represent a landmark contribution to clinical practice standardisation.
(4) Structured MDT protocols and implementation science: research comparing different MDT collaboration models, identifying minimum team composition and protocol requirements, and examining implementation barriers and facilitators across different healthcare system contexts would translate existing evidence into scalable clinical improvement.
(5) LMIC-specific tracheostomy rehabilitation research and PROM development: dedicated implementation science and clinical research in LMIC settings—developing and validating adapted protocols that account for resource constraints, workforce capacity, and local healthcare system realities—is urgently needed alongside the development and validation of PROMs specifically designed for tracheostomized PMV patients, covering voice quality, swallowing function, psychological well-being, social participation, sleep quality, and return to meaningful activities.

## 6. Conclusions

Prolonged mechanical ventilation and tracheostomy impose a substantial and compounding burden on patients—physiological, psychological, and social—that extends far beyond the ICU admission and persists long after hospital discharge. Successful decannulation, when achieved, represents not merely the removal of an artificial airway but the restoration of voice, swallowing, communication, and the capacity for meaningful social participation. For too many patients, this outcome remains unrealised—not because it is physiologically unachievable, but because the clinical systems, protocols, and multidisciplinary frameworks needed to achieve it reliably are absent or inconsistently applied.

This review has synthesised current evidence across the full spectrum of respiratory rehabilitation for tracheostomized PMV patients—from the patient-level factors that shape the rehabilitation trajectory, through six core intervention domains that build decannulation readiness, to the structured assessment and stepwise process of decannulation itself. High-intensity IMT at no less than 50% of MIP is the most evidence-supported single intervention and should be initiated early in all patients without contraindication. Expiratory muscle training remains critically understudied despite its direct relevance to cough efficacy and decannulation success, and must be prioritised in future research. Nutritional optimisation should be considered an active rehabilitation intervention rather than purely supportive care. Light sedation and the ABCDEF bundle are important enablers of active rehabilitation participation. Speaking valve use is an integral component of the decannulation pathway. Structured MDT collaboration guided by institution-level protocols should be the standard of care. The absence of unified, evidence-based decannulation guidelines is one of the most consequential gaps in the field, as illustrated by decannulation rates as low as 22% in some general ICU cohorts—though rates of 40–70% are reported in specialised weaning centres—highlighting the influence of institutional protocols and system-level factors on outcomes.

Two broader themes transcend any individual intervention. First, the definition of treatment success must be expanded beyond survival and decannulation to encompass the full spectrum of patient-reported outcomes—voice restoration, psychological well-being, and social reintegration. Physiological success that does not translate into meaningful functional recovery is an incomplete outcome. Second, the global inequity in tracheostomy rehabilitation care—where evidence-based interventions available in high-income settings are inaccessible to the majority of the world’s tracheostomized patients—represents both a moral imperative and a research priority. This review provides clinicians, rehabilitation specialists, and multidisciplinary teams with a practical, evidence-informed framework for optimising respiratory rehabilitation and achieving successful decannulation in tracheostomized PMV patients. Its ultimate measure of success will be whether the tracheostomized patient whose clinician reads these words achieves the voice, the swallow, and the return to life that successful decannulation makes possible.

## Data Availability

No new data were created or analyzed in this study.

## Supplementary Materials

The following supporting information can be downloaded at: https://www.mdpi.com/article/10.3390/healthcare14121804/s1. Supplementary Table S1. Reasons for exclusion of 36 full-text articles that did not meet eligibility criteria after full-text review. Supplementary Table S2. Evidence strength supporting key rehabilitation and decannulation thresholds, including evidence tier classification (Tier 1: RCTs/multicentre prospective studies; Tier 2: observational cohort studies/clinical guidelines; Tier 3: single-centre studies/expert consensus/exploratory findings), source population, and validation status in tracheostomized PMV patients. Supplementary Table S3. Summary of evidence tier labels applied to each key intervention in the manuscript, organised by section number. Supplementary Table S4. Summary of key recommendations in this review, their source populations, and whether they are derived directly from tracheostomized PMV populations or extrapolated from adjacent populations. Supplementary Table S5. Detailed summary of the 51 included studies, including study design, population, sample size, intervention/exposure, primary outcome, key finding, and limitation for each study.

## Abbreviations

AARC	American Association for Respiratory Care
ABCDEF	ICU liberation bundle
ACBT	Active Cycle of Breathing Techniques
ACV	Above Cuff Vocalisation
ALS	Amyotrophic lateral sclerosis
ARDS	Acute respiratory distress syndrome
ASPEN	American Society for Parenteral and Enteral Nutrition
CMV	Controlled mechanical ventilation
COPD	Chronic obstructive pulmonary disease
DTF	Diaphragmatic thickening fraction
EDP	External diaphragm pacing
EMT	Expiratory muscle training
EN	Enteral nutrition
ESPEN	European Society for Clinical Nutrition and Metabolism
FICM	Faculty of Intensive Care Medicine
FiO_2_	Fraction of inspired oxygen
GRADE	Grading of Recommendations, Assessment, Development and Evaluation
GTC	Global Tracheostomy Collaborative
HIC	High-income countries
ICS	Intensive Care Society
ICU	Intensive care unit
IMT	Inspiratory muscle training
LMIC	Low- and middle-income countries
MDT	Multidisciplinary team
MeSH	Medical Subject Headings
MIP	Maximal inspiratory pressure
MV	Mechanical ventilation
NAMDRC	National Association for Medical Direction of Respiratory Care
NIPPV	Non-invasive positive pressure ventilation
NIV	Non-invasive ventilation
OSA	Obstructive sleep apnea
PaCO_2_	Partial pressure of arterial carbon dioxide
PaO_2_	Partial pressure of arterial oxygen
PaO_2_/FiO_2_	Oxygenation index
PCF	Peak cough flow
PDT	Percutaneous dilatational tracheostomy
PEEP	Positive end-expiratory pressure
PMV	Prolonged mechanical ventilation
PROSPERO	International prospective register of systematic reviews
PROM	Patient-reported outcome measure
PSV	Pressure support ventilation
QoL	Quality of life
RCT	Randomised controlled trial
RICU	Respiratory intensive care unit
RR	Respiratory rate
RT	Respiratory therapist
SANRA	Scale for the Assessment of Narrative Review Articles
SBT	Spontaneous breathing trial
SLP	Speech–language pathologist
SpO_2_	Peripheral oxygen saturation
SWiM	Synthesis Without Meta-analysis
TCM	Traditional Chinese medicine
VAP	Ventilator-associated pneumonia
VCO_2_	Carbon dioxide production
VIDD	Ventilator-induced diaphragmatic dysfunction
WIND	Weaning according to a New Definition

## Appendix A. Full PubMed Search String

*(tracheostomy[MeSH Terms] OR tracheotomy[MeSH Terms] OR tracheostom*[Title/Abstract] OR tracheotom[Title/Abstract])
*AND*
*(respiration, artificial[MeSH Terms] OR ventilator weaning[MeSH Terms] OR prolonged mechanical ventilation[Title/Abstract] OR PMV[Title/Abstract] OR difficult weaning[Title/Abstract])*
*AND*
*(rehabilitation[MeSH Terms] OR physical therapy modalities[MeSH Terms] OR respiratory therapy[MeSH Terms] OR deglutition[MeSH Terms] OR speech-language pathology[Title/Abstract] OR inspiratory muscle training[Title/Abstract] OR decannulation[Title/Abstract] OR weaning[Title/Abstract])*
*Filters applied: Publication date—2019/05/01 to 2026/02/15; Languages—English, Chinese; Species—Humans*
*Last search date: 15 February 2026*
