# Supplementary material for: Respiratory Rehabilitation and Decannulation in Adults with Prolonged Mechanical Ventilation After Tracheostomy: A Narrative Review
Source: Healthcare (Basel). 2026 Jun 22;14(12):1804. doi: 10.3390/healthcare14121804 (PMC13299066; doi:10.3390/healthcare14121804)
Supplement: Supplementary file 1 [file healthcare-14-01804-s001.zip › healthcare-4326214-supplementary.pdf]

Supplementary File S1

Full PubMed Search String

(tracheostomy[MeSH Terms] OR tracheotomy[MeSH Terms] OR tracheostom[Title/Abstract] OR tracheotom[Title/Abstract])

AND

(respiration, artificial[MeSH Terms] OR ventilator weaning[MeSH Terms] OR prolonged mechanical ventilation[Title/Abstract] OR PMV[Title/Abstract] OR difficult weaning[Title/Abstract])

AND

(rehabilitation[MeSH Terms] OR physical therapy modalities[MeSH Terms] OR respiratory therapy[MeSH Terms] OR deglutition[MeSH Terms] OR speech-language pathology[Title/Abstract] OR inspiratory muscle training[Title/Abstract] OR decannulation[Title/Abstract] OR weaning[Title/Abstract])

Filters applied: Publication date — 2019/05/01 to 2026/02/15; Languages — English, Chinese; Species — Humans

Last search date: 15 February 2026

Supplementary Table S1 — Reasons for Exclusion of Full-Text Articles (n = 30)

No.	Reason for Exclusion	Number of Studies
**1**	Population not tracheostomised PMV adults (paediatric populations, short-term ventilation only, general ICU without tracheostomy)	8
**2**	Intervention not related to respiratory rehabilitation or decannulation (surgical technique studies, unrelated pharmacological trials)	6
**3**	Outcomes not relevant to decannulation, weaning, or respiratory rehabilitation	5
**4**	Insufficient data or non-extractable outcomes (abstract only, conference poster, incomplete dataset)	3
**5**	Duplicate population or overlapping dataset from an already-included study	2
**6**	Published outside the defined date range (before May 2019)	3
**7**	Published in a language other than English or Chinese	9
**Total**		**36**

Supplementary Table S2

Evidence Strength Supporting Key Rehabilitation and Decannulation Thresholds:

Parameter	Threshold	Evidence Tier	Source Population	Validated in PMV Tracheostomy Patients?
**IMT intensity**	≥50% MIP	Tier 1–2	General ICU; non-tracheostomy PMV	Not specifically validated
**Peak cough flow**	>160 L/min	Tier 2–3	Neuromuscular disease; spontaneously breathing tracheostomy adults	Not prospectively validated in heterogeneous PMV
**Suctioning frequency**	≤4 times/24 h	Tier 3	General tracheostomy cohorts	Not validated

**Gas exchange adequacy**	$PaCO_2$ <50 mmHg	Tier 2–3	General ventilator weaning literature	Indirect evidence only
**Tube capping duration**	24–48 hours continuous	Tier 2–3	Mixed tracheostomy populations	Not validated in heterogeneous PMV

Supplementary Table S3

Specific Tier labels to add in the manuscript at the point of each intervention:

Section	Intervention	Label to add
**4.1.1**	IMT ≥50% MIP	*[Tier 1–2]*
**4.1.2**	EMT	*[Tier 3 — insufficient evidence]*
**4.1.3**	Electroacupuncture	*[Tier 3, exploratory]*
**4.1.3**	External diaphragm pacing	*[Tier 3, exploratory]*
**4.2.1**	Early exercise training	*[Tier 2]*
**4.2.2**	Nutritional targets ESPEN/ASPEN	*[Tier 2, indirect evidence]*
**4.3**	ABCDEF bundle	*[Tier 1–2]*
**4.4**	Speaking valve use	*[Tier 2, indirect evidence]*
**4.5**	Airway complication management	*[Tier 2–3]*
**4.6**	PSV over CMV	*[Tier 2]*
**4.6**	Daily SBT screening	*[Tier 1]*
**5.1.2**	PCF >160 L/min	*[Tier 2–3, indirect evidence]*
**5.1.2**	Suctioning ≤4 times/24h	*[Tier 3]*
**5.1.2**	$PaCO_2$ <50 mmHg	*[Tier 2–3]*

Summary Table S4

The following table summarises which key recommendations in this review are derived directly from tracheostomized PMV populations and which are extrapolated from adjacent populations.

Recommendation	Source Population	Direct or Extrapolated
**IMT ≥50% MIP**	General ICU; non-tracheostomy PMV	Extrapolated
**EMT for cough efficacy**	Insufficient evidence in any population	Evidence gap
**Early exercise rehabilitation**	Mixed ICU including PMV	Partially direct

**Nutritional targets 1.3 g/kg/day**	General critically ill patients	Extrapolated
**ABCDEF bundle for sedation**	General ICU patients	Extrapolated
**Speaking valve physiological benefits**	Post-stroke; acquired brain injury	Extrapolated
**ACV for voice restoration**	Mixed tracheostomy ICU patients	Partially direct
**PCF >160 L/min decannulation threshold**	Neuromuscular disease; spontaneously breathing tracheostomy adults	Extrapolated
**Suctioning ≤4 times/24h**	General tracheostomy cohorts	Partially direct
**Instrumental swallowing assessment**	Post-stroke; acquired brain injury	Extrapolated
**Fiberoptic bronchoscopy before decannulation**	Mixed tracheostomy case series	Partially direct
**$PaCO_2$ <50 mmHg**	General ventilator weaning literature	Extrapolated
**Capping trial 24–48 hours**	Mixed tracheostomy populations	Partially direct
**NIPPV bridge for neuromuscular patients**	Neuromuscular respiratory failure	Direct — neuromuscular only
**Early tracheostomy within 10 days**	Severe burns; trauma ICU	Extrapolated

Supplementary Table S5 — Summary of Included Studies

#	Author (Year)	Study Design	Population	Sample Size	Intervention / Exposure	Primary Outcome	Key Finding	Limitation
**1**	Bissett et al. (2019)	Narrative review / practical guide (with clinical experience)	ICU patients requiring invasive mechanical ventilation for ≥7 days (ventilator-dependent or recently weaned)	N/A	Inspiratory muscle training (IMT) using threshold device; high-intensity low-repetition protocol (5 sets of 6 breaths at ≥50% MIP, increased daily)	Inspiratory muscle strength, weaning outcomes, quality of life	IMT is safe and feasible in selected ICU patients; improves inspiratory muscle strength and quality of life; may enhance weaning success	Narrative review / practical guide — no primary data; optimal training parameters not established; based on single-centre experience
**2**	Gallice et al. (2023)	Prospective single-centre non-randomized cohort study	Tracheostomized acquired brain injury (ABI) patients weaned from mechanical ventilation, after ICU discharge	30	Pluridisciplinary tracheostomy weaning protocol (5-step logigram: cuff deflation, manual occlusion, speaking valve for 12 h, plug capping for 24 h, then decannulation) guided by patient-specific stability parameters	Decannulation success (no recannulation within 96 h), tracheostomy weaning duration	Decannulation rate 90% (26/30) with 100% success (no recannulations); mean weaning duration 7.6 days (SD 4.6); protocol appears safe outside ICU without routine instrumental assessment	Small sample size (n=30), single-centre, no control group, lack of gold-standard instrumental evaluation (FEES/VFSS), one death possibly related to protocol

**3**	Paul et al. (2025)	Secondary analysis of a cluster-randomized controlled trial	Critically ill patients receiving prolonged mechanical ventilation (PMV: ≥21 days invasive MV or ≥4 days via tracheostomy) in 3 German ICUs	90	Prolonged mechanical ventilation (stratified by weaning success within 6 months after ICU discharge)	6-month mortality, care place transitions, hospital readmissions, and health-related quality of life (EQ-5D-5L)	46% died; among 69 discharged alive, 75% were weaned; successfully weaned patients had more care transitions, more time at home, better quality-adjusted life days, and lower 6-month mortality (17% vs 65%) than unsuccessfully weaned patients	Secondary analysis with small sample size (n=90) from two academic clusters in one metropolitan area, limiting external validity; no pre-ICU HrQoL; possible loss-to-follow-up bias
**4**	Piraino et al. (2022)	Clinical practice guideline (systematic review + modified RAND/UCLA consensus)	Hospitalized adults in acute care settings requiring supplemental oxygen	N/A	Oxygen therapy management: oxygenation targets (SpO2 ranges), high-flow nasal cannula (HFNC) vs conventional oxygen, timing of HFNC initiation, humidification	Recommendations for SpO2 targets, HFNC use, and humidification to improve length of stay, mortality, escalation of care, re-intubation, and patient comfort	Recommended SpO2 94–98% (88–92% for COPD); early HFNC may avoid NIV/intubation; HFNC postextubation reduces re-intubation; no benefit of HFNC in immunocompromised; humidification for flows >4 L/min improves comfort	Clinical practice guideline — no primary data; evidence levels B and C (weak to moderate); many recommendations based on expert consensus rather than high-quality RCTs; limited evidence for some PICO questions
**5**	Devlin et al. (2024)	Pre-post quality improvement study (before-after intervention)	Adult patients with tracheostomy in acute care setting at a community-based hospital	203 (94 pre-intervention, 109 post-intervention)	Implementation of AARC clinical practice guidelines: interdisciplinary tracheostomy team, weekly bedside rounding, standardized order sets, care bundles, weaning/decannulation protocols, and staff education	Decannulation rate (overall and in non-COVID acutely tracheostomized survivors), SLP consults, one-way speaking valve consults, hospital/ICU LOS, ventilator days, tracheostomy mask days, mortality, 30-day readmissions, time to decannulation	Significant increases post-intervention: decannulations in non-COVID acutely tracheostomized survivors (11.8% → 33.3%, p=0.043), SLP consults (53.2% → 89.0%, p<0.001), speaking valve consults (17.0% → 32.1%, p=0.02). No significant changes in LOS, mortality, or readmissions.	Single-centre, pre-post design without control group; confounding from COVID-19 pandemic; manual data validation; may not generalise to larger tertiary centres or those with existing tracheostomy teams
**6**	Lippi et al. (2022)	Systematic review and meta-analysis of RCTs	Acute critically ill patients receiving mechanical ventilation in ICU, without pre-existent chronic respiratory conditions	12 RCTs (total N=791; 413 intervention, 378 control)	Comprehensive physiotherapy interventions (early mobilization, positioning, airway clearance techniques, lung expansion, respiratory muscle	Mechanical ventilation duration, extubation time, weaning time	Comprehensive physiotherapy significantly reduced MV duration by 3.23 days (95% CI −5.79 to −0.67, p=0.01); improved weaning success and respiratory muscle strength	Systematic review/meta-analysis — high heterogeneity across studies; could not assess efficacy of single rehabilitation modalities; optimal rehabilitation program remains uncertain;

					training, automatic weaning systems)			limited long-term outcome data
**7**	Tarride et al. (2025)	Trial-based economic evaluation (cost-effectiveness analysis alongside RCT)	Adult ICU patients receiving invasive mechanical ventilation (mean age 61.5 years, 56.9% male)	360 (178 cycling + usual physiotherapy, 182 usual physiotherapy alone)	Early in-bed cycle ergometry (30 min/day on weekdays, started within first 4 days of MV) added to usual physiotherapy vs usual physiotherapy alone	Differences in costs (2024 Canadian dollars) and quality-adjusted life-years (QALYs) at 90 days	Per-patient cycling cost CA$321 (0.5% of index hospitalization costs); no significant differences in 90-day costs (difference CA$5841, 95% CI –7666 to 18797) or QALYs (difference –0.0009, 95% CI –0.0185 to 0.0182); probability of cost-effectiveness at CA$50,000/QALY = 0.19	Trial-based economic evaluation — no significant clinical or cost-effectiveness differences; missing data imputed; unit costs from Ontario only; follow-up resource use based on patient recall; results may not generalise to centres with lower usual physiotherapy levels
**8**	Wu et al. (2025)	Prospective randomized controlled trial	Adult ICU patients receiving invasive mechanical ventilation via endotracheal tube, stable/recovery phase, conscious (RASS 0–2 or GCS 13–15), on VC+PS mode	159 (78 control, 81 intervention; 170 enrolled, 11 dropped out)	Early off-bed activity (patient transfer device to wheelchair followed by fist clenching, arm lifting, elastic band chest expansion, cycling, standing, stepping/walking) vs conventional progressive early mobilization (bed-based passive to active exercises, eventually sitting/standing)	Respiratory mechanics (MV, MVi, MVe, RR, Raw, Cdyn, WOB, Pm, PIP, Pplat, driving pressure, PEEP) at baseline and 5,10,15,30 min post-intervention; duration of invasive MV, ICU LOS, 48-h reintubation rate	Early off-bed activity significantly improved MV, MVi, MVe, RR, Cdyn and reduced Raw, WOB vs control (time, intervention, interaction effects $p<0.01$); no differences in airway pressures; shortened MV duration (84.2 vs 121.9 h) and ICU LOS (5.2 vs 7.1 d); no difference in 48-h reintubation rate	Single-centre, small sample size, no blinding (intervention not possible), short-term outcomes only (30 min post-intervention), underpowered for reintubation
**9**	Rose and Messer (2024)	Narrative review	Adult critically ill patients requiring prolonged mechanical ventilation (PMV) and/or difficult weaning	N/A	Various management strategies including weaning methods, tracheostomy timing and cuff management, physiotherapy (mobilization, airway clearance, inspiratory muscle training), nutrition, occupational therapy, psychological services, and	Overview of definitions, prevalence, outcomes, and evidence for interventions in PMV patients	Approximately 5-10% of critically ill adults require PMV with 1-year mortality ~60% and only 19% return home; evidence for weaning protocols, tracheostomy timing, rehabilitation, and nutrition specific to PMV is limited; a structured, individualized, interprofessional approach is recommended	Narrative review — no primary data; not systematic; evidence summary based on selected literature

					specialized weaning centers			
**10**	Rosero et al. (2021)	Narrative review	Adult patients with tracheostomy presenting for surgical or diagnostic procedures under general anesthesia or sedation/analgesia	N/A	Intraoperative airway management considerations for tracheostomy patients (including tracheostomy device types, exchange with endotracheal tube, management of T-tube, accidental decannulation emergencies)	N/A (narrative review)	Airway management of tracheostomy patients requires understanding of tracheostomy devices, stoma maturity, indication for tracheostomy, and patient positioning; multidisciplinary care and preprocedure checklist recommended; algorithm for accidental decannulation provided	Narrative review — no primary data
**11**	Weidlich et al. (2023)	Scoping review	Adults (≥18 years) with temporary or permanent tracheostomy, living at home, partially or fully self-sufficient in tracheostomy care (non-ventilated)	34 studies (24 quantitative, 8 qualitative, 2 mixed methods)	Self-management tasks and skills (managing therapeutic regimen, tracheostomy-related physical/functional changes, changed roles, changed everyday behavior, managing emotions)	Identification and modelling of self-management dimensions and sub-dimensions for tracheostomy patients in home setting	Three self-management dimensions identified: managing therapeutic regimen (82% of studies), managing role and behavior changes (79%), managing emotions (47%); developed theoretical model with patient at centre; most studies focused on laryngectomy patients (91%)	Scoping review — no formal quality assessment; limited to four databases (Medline, CINAHL, PsycINFO, Cochrane) and English/German; single reviewer for selection/analysis; exclusion of low-income countries may limit generalisability
**12**	Brown (2022)	Narrative review	Patients with tracheostomy (especially neuromuscular disorders) transitioning from acute care to home, including those requiring prolonged mechanical ventilation	N/A	Care transitions (ICU → LTAC/rehabilitation → home), transition from tracheostomy to noninvasive positive pressure ventilation (NIPPV), ventilator device selection, decannulation decisions	Overview of transition processes, ventilator weaning, decannulation, and home care for tracheostomy patients	Patients undergo ~4 care transitions; early vs late tracheostomy timing shows no difference in ventilator days or ICU LOS; peak cough flow >160 L/min predicts successful decannulation to NIPPV; recommendations for home transition include 24-h caregiver training and emergency bag	Narrative review — no primary data; publication year not specified in the provided file
**13**	Wiemann et al. (2022)	Cross-sectional retrospective chart review	Ventilator-dependent patients with COVID-19 undergoing tracheotomy at a single academic medical center	50	Tracheotomy procedure	Analgesic and sedative requirements (oral morphine equivalents [OME], lorazepam equivalents,	Significant decreases post-tracheotomy: mean OME ↓49.4 mg (p=0.014), lorazepam equivalents ↓45.1 mg (p=0.031),	Small sample size, single-centre, retrospective design, excludes patients on ECMO or with

						dexmedetomidine dose, propofol dose) at 24 h pre-tracheotomy vs 48 h post-tracheotomy	dexmedetomidine ↓0.34 µg/kg/h (p=0.012), propofol ↓20.5 µg/kg/min (p<0.0001)	opioid/benzodiazepine dependence
**14**	Zhang et al. (2024)	Retrospective observational cohort study	Tracheostomized stroke patients (age ≥60–80 years, first onset, stable vital signs, with pulmonary infection)	200 (100 EDP + electroacupuncture, 100 EDP alone)	Extrinsic diaphragmatic pacing (EDP) + electroacupuncture at back-shu acupoints (Lung Shu, Spleen Shu, Kidney Shu) vs EDP alone; both groups received standard care; treatment 5×/week for 4 weeks	Cough reflex score, clinical pulmonary infection score, blood gas parameters (pH, PaO_2, $PaCO_2$, OI), diaphragmatic function (thickness, mobility, contraction speed), lung function (FVC%, FEV1%), MIP, MEP, extubation time and success rate	EDP + electroacupuncture group had higher total effective rate (91% vs 80%, p<0.05), lower cough reflex and infection scores, better blood gas, greater diaphragm thickness/mobility/contraction speed, higher FVC%/FEV1%/MIP/MEP, shorter extubation time (21.3 vs 27.1 d, p<0.001), and higher extubation success rate (70% vs 57%, p=0.002) vs EDP alone	Retrospective observational design (not randomized), single-centre, small sample size, no long-term follow-up, potential selection bias and confounding
**15**	Filoni et al. (2025)	Scoping review	Patients with tracheostomy or tracheal cannula (mixed etiologies: malignant lesions, prolonged MV, head/neck cancer, neuromuscular disorders, ARDS, traumatic brain injury)	13 studies	Adjustment disorders (anxiety, depression, stress) following tracheostomy	Impact of adjustment disorders on quality of life (QoL), functional status, rehabilitation outcomes, extubation time	Adjustment disorders (anxiety, depression, stress) are common after tracheostomy and significantly impair QoL, prolong recovery/extubation time, and affect rehabilitation; communication difficulties are a major source of stress; structured interventions (communication aids, educational programs, quality improvement) can reduce anxiety/depression	Scoping review — no quality assessment; high heterogeneity across studies (different populations, outcome measures, lack of control groups); limited long-term data; possible publication and language bias
**16**	McMahon et al. (2023)	Single-centre retrospective observational cohort study	Consecutive ICU patients requiring tracheostomy insertion (excluding permanent tracheostomy prior to admission) at a university-affiliated quaternary referral hospital	304	Tracheostomy insertion (analysed by early vs late timing [<10 vs ≥10 days] and age category [≤65 vs ≥66 years])	Patient-centred outcomes: time to non-invasive ventilation (NIV), ventilator-free breathing (VFB), speaking valve placement, swallow assessment, mobilization (dynamic sitting, sit to edge, sit to stand), sedation days,	Tracheostomy facilitated rapid sedation weaning (median 0 days post-tracheostomy) and ventilation weaning (94% to NIV by day 1, 72% to VFB by day 5); early tracheostomy associated with shorter ICU LOS and faster weaning; older patients (≥66) had higher mortality (36.1%) but two-thirds achieved	Single-centre, retrospective design, no control group (surrogate for extubation), heterogenous case mix, follow-up only to hospital discharge

						ICU/hospital LOS, mortality, discharge disposition	meaningful outcomes (VFB, speaking valve, swallow, sitting); 40.9% of survivors discharged home	
**17**	Smailes et al. (2022)	Service evaluation (prospective early tracheostomy cohort vs retrospective late tracheostomy cohort)	Adult ICU patients with severe burns requiring prolonged respiratory support	41 (19 early tracheostomy, 22 late tracheostomy)	Early tracheostomy (≤10 days after admission) vs late tracheostomy (>10 days)	Days of mechanical ventilation, first day of active exercise after admission, ICU/hospital length of stay, Functional Assessment for Burns (FAB) score at ICU and hospital discharge, discharge disposition	Early tracheostomy associated with fewer ventilation days (16 vs 33, p=0.001), earlier active exercise (day 8 vs 25, p<0.0001), shorter hospital LOS (65 vs 88 days, p=0.018), and higher FAB score at hospital discharge (32 vs 28, p=0.016) compared to late tracheostomy	Small sample size (n=41), before-after design with potential temporal bias, single-centre, non-randomized, lack of multivariate analysis
**18**	Sutt et al. (2020)	Prospective observational cohort study (multistate modelling)	Adult ICU patients requiring tracheostomy (tertiary referral centre, mixed diagnoses: cardiorespiratory, respiratory, sepsis)	276	Tracheostomy placement (timing analysed as intermediate event; comparison of outcomes before vs after tracheostomy)	Time to patient-centric outcomes (verbal communication using speaking valve, oral intake, out-of-bed exercises); analgesic, sedative, and antipsychotic drug doses; ICU and long-term mortality; mobility score on ICU discharge	After tracheostomy, patients achieved verbal communication 7.4 d earlier (95% CI −9.1 to −4.9), oral intake 7.0 d earlier (95% CI −10 to −4.6), and out-of-bed exercises 6.2 d earlier (95% CI −8.4 to −4) compared to patients without tracheostomy; significantly less analgesics and sedatives, more antipsychotics; no significant mortality difference	Single-centre, observational, no randomisation; patient cohort may not generalise; no routine delirium measurement; long-term quality-of-life data not collected
**19**	Han et al. (2024)	Systematic review and meta-analysis of RCTs	Adult patients receiving mechanical ventilation in critical care units (mixed and specialised ICUs)	21 RCTs (N=3,621 patients; 1,796 early, 1,825 late tracheotomy)	Early tracheotomy (≤10 days after intubation/ICU admission) vs late tracheotomy (>10 days or later definitions per study)	Mortality, incidence of ventilator-associated pneumonia, duration of mechanical ventilation, length of ICU stay, length of hospital stay	Early tracheotomy significantly reduced mechanical ventilation duration (MD −2.77 days; 95% CI −5.10 to −0.44) and ICU length of stay (MD −6.36 days; 95% CI −9.84 to −2.88), but did not significantly reduce mortality (RR 0.86; 95% CI 0.73–1.00; p=0.06) or pneumonia (RR 0.86; 95% CI 0.74–1.01; p=0.06)	Systematic review/meta-analysis — substantial heterogeneity among RCTs; variable definitions of early/late tracheotomy; mostly specialised ICU patients (not general ICU); lack of uniform pneumonia diagnostic criteria; sensitivity analyses showed some results not robust

**20**	Rossi et al. (2025)	Systematic review	Adult patients with acute respiratory distress syndrome (ARDS) requiring mechanical ventilation in ICU	20 studies (N=4,022 patients with ARDS who underwent tracheostomy)	Tracheostomy (including comparison of early vs late timing where reported)	ICU length of stay (primary); hospital length of stay, duration of mechanical ventilation, mortality, tracheostomy-related adverse events	Mean ICU LOS 30.2 days, mean MV duration 27 days, hospital mortality 38.4%; tracheostomy-related complications generally minor (local bleeding most common); no clearly defined impact on ICU LOS due to study variability	Systematic review — high heterogeneity across studies; most studies focused on COVID-19 ARDS limiting generalisability; majority observational with moderate risk of bias; lack of standardised weaning protocols
**21**	Davis et al. (2021)	Retrospective case series	Adult patients who underwent tracheostomy at an urban level 1 trauma center	255	Speech language pathology (SLP) evaluation (general evaluation, speaking valve, swallow study) after tracheostomy	Rate of SLP evaluation after tracheostomy; secondary: speaking valve and swallow study utilization, downsize/decannulation rates, duration of cannulation, complications	77.3% received SLP evaluation (mean 5.9 days post-surgery); 33.7% received speaking valve, 52.9% swallow study; SLP evaluation associated with higher downsizing (87% vs 53%) and decannulation rates (68% vs 43%); head/neck cancer, trauma, successful swallow study increased odds of decannulation; obesity and prior tracheostomy decreased odds	Retrospective, single-centre; SLP consults often placed for more complex patients (selection bias); no standardised protocol; external validity limited
**22**	Silveira et al. (2024)	Pre-post quality improvement study (retrospective historical control vs post-intervention cohort)	Adult tracheostomized patients in trauma ICU of a Level 1 trauma hospital (traumatic brain injury, spinal cord injury, polytrauma)	91 (36 pre-intervention, 55 post-intervention)	SLP-led multidisciplinary tracheostomy team (twice-weekly rounding, standardized protocols/order sets, education, visual signage)	SLP consultation rate, days to SLP consult, Passy-Muir Valve (PMV) use, oral diet initiation, downsizing, decannulation, hospital LOS	Post-intervention: SLP consultation increased (58.3%→89.1%, p<0.001; median days 6→2, p<0.001), PMV use increased (44.4%→67.3%, p=0.031), decannulation rate increased (8.3%→30.9%, p=0.011); oral diet, downsizing, LOS showed non-significant improvements	Single-centre, pre-post design (historical control, no concurrent control), trauma-specific population limits generalisability, potential confounding from unmeasured variables (injury severity, comorbidities), missing data, no cost analysis
**23**	Khanum et al. (2022)	Cross-sectional observational study (questionnaire-based)	Healthcare professionals (doctors and nurses) working in medicine/surgery wards, ER, and ICUs of four tertiary care hospitals in Karachi, Pakistan	254	Knowledge assessment questionnaire on tracheostomy care and management of early complications	Knowledge score (percentage of correct answers) regarding tracheostomy suctioning, cuff management, tube	52% of participants had good knowledge (>50% correct); areas of poorest knowledge: adequate cuff pressure (38.9%), suction pressure (39.4%), response to tube blockage (31.1%), stay suture	Cross-sectional design with convenience sampling; volunteer bias; higher proportion of male participants; theoretical assessment only (no

						blockage, feeding, complications	removal time (34.8%), earliest sign of stomal infection (31.5%)	simulation or practical evaluation)
**24**	Abu-Sahayoun et al. (2023)	Cross-sectional descriptive study	Critical care nurses working in CCUs (cardiac, thoracic, surgical, medical, general, long-term) of four government hospitals in Jordan	260	Knowledge assessment using structured questionnaire (38 items, six dimensions: general trachea info, tracheostomy, tracheostomy tube, tracheostomy care, suctioning, nursing responsibility)	Level of knowledge (low <60%, moderate 60–80%, high >80% correct) regarding tracheostomy care	Overall knowledge moderate (mean 26.05/38, 68.6%); high knowledge only in "tracheostomy care" dimension; significant associations with age, educational level, work experience ($p<0.001$); gender significantly associated only with suctioning dimension ($p=0.001$, females higher)	Cross-sectional design, convenience sampling, self-report bias, single country (Jordan), results may not generalise to other hospitals or settings
**25**	Selekwa et al. (2023)	Scoping review	Tracheostomy care in low- and middle-income countries (LMICs) — includes healthcare providers, caregivers, and patients	N/A (18 studies included)	Quality improvement interventions for tracheostomy care (needs assessments, caregiver training programs, home-based or hospital-based interventions, standardised protocols)	Summary of published tracheostomy care and quality improvement studies in LMICs, highlighting challenges, barriers, and gaps	Only 18 studies identified across 10 countries; unique challenges include language/literacy barriers, resource limitations (running water, electricity, equipment), financial burden on families, and lack of standardised care; opportunities exist for needs assessments, tailored educational materials, mobile health interventions, and use of implementation science frameworks	Scoping review — no primary data; possible publication bias (English-only, unpublished programs not captured); high heterogeneity across studies limits comparison; may not fully represent all LMIC settings
**26**	Brenner et al. (2025)	Cross-sectional descriptive study (global multi-stakeholder survey)	Healthcare professionals (speech-language pathologists, nurses, respiratory therapists, physicians), patients, and family caregivers from the Global Tracheostomy Collaborative learning community (14 countries)	170 respondents	19-item survey on tracheostomy care challenges (education, workforce competency, access, affordability, caregiver empowerment) rated on 0–10 severity scale plus qualitative free-text	Median severity scores for challenges; qualitative themes on suctioning, tube changes, stoma care, barriers, access, affordability	Most critical issues: limited HCP with tracheostomy expertise (median 8), access for underserved communities (7), affordability (6); key themes: inadequate standardised training, variation in suctioning technique, fear of tube changes, lack of tracheostomy teams, insufficient caregiver empowerment, financial strain, geographic barriers	Cross-sectional survey; sample predominantly from high-income countries (US, UK, Australia) limiting generalisability to low-resource settings; self-selection/response bias; imbalanced stakeholder groups; no statistical comparisons due to small caregiver sample

**27**	McCool et al. (2020)	Prospective randomized controlled trial	Mechanically ventilated ICU patients (>48 h) ready for spontaneous breathing trial (PEEP <8 cmH_2O, FiO_2 <50%, SpO_2 >92%, minimal sedation)	32 (15 control, 17 intervention)	Usual care + diaphragm ultrasound (Δtdi% = percent change in diaphragm thickness from end-expiration to end-inspiration) vs usual care alone	Time from ultrasound to extubation (hours)	In patients with Δtdi% ≥30%, time to extubation was significantly shorter in intervention vs control (4.8 ± 8.4 vs 35.0 ± 41.0 h, p=0.04); overall time to extubation shorter in those with Δtdi% ≥30% vs <30% (23.2 ± 35.2 vs 57.3 ± 52.0 h, p=0.046); sensitivity 90.9%, specificity 86.7% for extubation success at 24 h using Δtdi% ≥30%	Small sample size, single ultrasonographer limiting generalisability
**28**	Bissett et al. (2020)	Narrative review (expert review)	ICU patients with prolonged mechanical ventilation	N/A	Respiratory muscle rehabilitation (inspiratory muscle training, threshold loading, high-intensity training)	N/A (narrative review; covers weaning duration, inspiratory muscle strength, quality of life, mortality)	Respiratory muscle weakness affects 63% of MV patients, associated with higher mortality and weaning failure; inspiratory muscle training improves strength, may reduce weaning duration by ~3 days, and improves quality of life; practical guidance for targeted training provided	Narrative review — no primary data; not systematic
**29**	Li et al. (2025)	Retrospective comparative cohort study	Severe stroke patients with tracheotomy (age 18–70 years, onset <6 months, ventilator use <16 h/day)	50 (25 ACBT alone, 25 ACBT + EDP)	Active cycle of breathing techniques (ACBT) alone vs ACBT combined with external diaphragm pacing (EDP) for 6 weeks (30 min twice daily, 5 days/week)	Diaphragmatic excursion (DE) and thickening fraction (Tfdi) by ultrasound, PaO_2, $PaCO_2$, chest CT improvement, Borg and VAS dyspnea scores	ACBT + EDP significantly improved left/right DE and Tfdi, increased PaO_2, decreased $PaCO_2$, and reduced Borg/VAS scores vs ACBT alone at 3 and 6 weeks ($p<0.05$); chest CT improvement not significantly different	Retrospective design, single-centre, small sample size, no blinding, short-term follow-up (6 weeks)
**30**	Zhu et al. (2020)	Prospective randomized controlled trial	Post-stroke tracheotomy patients (first stroke, onset 10–60 days, stable, tracheostomy tube in situ, not on ventilator, age 30–80 years)	70 randomized (69 completed: 35 observation, 34 control)	Electroacupuncture at Tianding (LI17), Fengchi (GB20), and C3–C5 Jiaji points (continuous wave, 10–20 Hz, 30 min/day, 6 days/week for 4 weeks) plus "Xingnao Kaiqiao" acupuncture and	Diaphragmatic motility (excursion in mm) measured by ultrasound	Both groups improved diaphragmatic motility after 4 weeks ($p<0.01$); electroacupuncture group showed significantly greater improvement than control (mean change 3.73 mm vs 2.18 mm, $p<0.01$)	Single-centre, small sample, publication year not specified, no comparison with external diaphragm pacing or sham acupuncture, limited outcomes (only diaphragmatic excursion), short follow-up (4 weeks)

					breathing training vs "Xingnao Kaiqiao" acupuncture plus breathing training alone			
**31**	Dong et al. (2021)	Prospective randomized controlled trial	ICU patients requiring mechanical ventilation for >72 h	80 (39 rehabilitation, 41 control)	Early rehabilitation therapy (six levels of exercises: positioning, turning, bed exercises, sitting, standing, walking) vs standard care	Diaphragmatic excursion (DE) and diaphragm thickening fraction (DTF) measured by ultrasound after 3 days of rehabilitation; duration of ventilator use, intubation duration	Rehabilitation group had significantly higher DTF at day 4 (0.15 ± 0.06 vs 0.12 ± 0.05, p=0.008) and smaller decline in DTF (p=0.026); shorter ventilator duration (7.49 ± 2.59 vs 9.41 ± 5.32 days, p=0.045) and intubation duration (8.31 ± 2.80 vs 10.37 ± 5.32 days, p=0.037) than control	Small sample size, single-centre, no stratification by disease or baseline diaphragm function, lack of post-discharge follow-up
**32**	Singer et al. (2023)	Clinical practice guideline (systematic review + consensus, ESPEN)	Critically ill adult ICU patients staying >48 h (including mechanically ventilated, surgical, trauma, septic, obese)	N/A	Clinical nutrition therapy (enteral nutrition, parenteral nutrition) including timing, route, dose, composition, monitoring	N/A (guideline recommendations)	56 recommendations: early EN within 48 h preferred over delayed EN or early PN; avoid early full feeding (hypocaloric nutrition in early phase); use indirect calorimetry for energy targets; protein 1.3 g/kg/d progressively; monitor glucose and treat >10 mmol/L with insulin; prokinetics (erythromycin) for gastric feeding intolerance; monitor refeeding syndrome	Clinical practice guideline — no primary data; some recommendations based on low/very low evidence (Grade 0 or B); not all recommendations validated in specific tracheostomy/PMV populations
**33**	Viner Smith et al. (2024)	Narrative review	Critically ill patients with persistent critical illness (ICU length of stay >10 days)	N/A	Nutrition considerations including energy and protein metabolism, prescription, delivery, barriers, monitoring, and multidisciplinary team role	N/A (narrative review)	Persistent critical illness (ICU >10 days) affects ~5–16% of admissions but consumes ~19–45% of ICU bed days and 55% of MV days, with 1-year mortality ~59%; guidelines lack specific recommendations for this population; energy expenditure is elevated but variable; protein metabolism shifts over time (anabolic	Narrative review — no primary data; not systematic; evidence base for nutrition in persistent critical illness is limited

							resistance early, possible neutral balance after 30 days); cumulative nutrition deficits are a concern; monitoring weight, muscle mass, and biochemistry is important; multidisciplinary team involvement is essential	
**34**	Pearson and Patel (2020)	Narrative review	Mechanically ventilated critically ill patients (including those with ARDS)	N/A	Sedation management strategies (choice of sedatives, sedation protocols, light vs deep sedation, daily interruption, no sedation)	N/A (narrative review)	Light sedation early in mechanical ventilation, avoiding benzodiazepines (use propofol or dexmedetomidine), with daily interruption or nurse-protocolised algorithms, reduces delirium, shortens MV duration, ICU/hospital LOS, and mortality; deep sedation is associated with worse outcomes; even in severe ARDS, light sedation is recommended	Narrative review — no primary data
**35**	Mart et al. (2021)	Narrative review	Critically ill adult ICU patients (including mechanically ventilated)	N/A	Prevention and management of delirium (ABCDEF bundle: Assess pain, Both SAT and SBT, Choice of sedation, Delirium assessment, Early mobility, Family engagement; nonpharmacologic interventions)	N/A (narrative review)	Delirium affects >50% of ICU patients, associated with increased mortality, longer MV, cognitive impairment; antipsychotics (haloperidol, atypical) not recommended for routine prevention/treatment; ABCDEF bundle is cornerstone of management; dexmedetomidine may reduce delirium but evidence limited	Narrative review — no primary data
**36**	McGrath et al. (2020)	Expert consensus guidance (multidisciplinary, narrative review)	Healthcare workers and adult patients requiring tracheostomy during COVID-19 pandemic (with additional considerations for children)	N/A	Recommendations for safe tracheostomy care including timing, insertion techniques (open vs percutaneous), aerosol-generating procedure precautions,	N/A (guidance document)	Tracheostomy facilitates weaning from prolonged ventilation; infectivity likely low 10-14 days post-intubation (20-24 days post-symptom onset); negative SARS-CoV-2 test not required before tracheostomy;	Expert consensus guidance — no primary data; based on early pandemic evidence (April 2020); recommendations may evolve with emerging data

					personal protective equipment (PPE), post-procedural management (cuff care, humidification, suction, weaning, decannulation), and cohorting		open surgical preferred but percutaneous not contraindicated; pre-procedural apnoea test advised; neuromuscular blockade maintained; post-procedural care modified to minimise aerosol generation (e.g., closed suction, reduced routine interventions, cuffed non-fenestrated tube, HME filters)	
**37**	Han et al. (2022)	Randomized, single-blinded controlled trial	Tracheostomized patients with aspiration following acquired brain injury (ABI), non-ventilated	20 (11 PMV intervention, 9 non-PMV control)	Passy-Muir Tracheostomy and Ventilator Swallowing and Speaking Valve (PMV) for 2 weeks vs no PMV	Penetration-Aspiration Scale (PAS) score, subglottic pressure (via computational fluid dynamics), velopharyngeal maximal pressure (VP-Max), upper esophageal sphincter relaxation duration (UES-RD)	PMV intervention significantly improved VP-Max, UES-RD, and subglottic pressure, and reduced PAS score ($p<0.05$) compared to non-PMV control; subglottic pressure increased from 0.53 to 6.95 cmH_2O after PMV	Small sample size, short follow-up (2 weeks), lack of long-term outcomes, no control for confounders (smoking, GERD, COPD), no laryngoscopy/bronchoscopy to assess structural anatomic factors
**38**	Martin et al. (2021)	Randomized controlled feasibility trial	Awake adult patients (GCS ≥9, CAM-ICU negative, RASS –1 to +1, English-speaking) undergoing percutaneous tracheostomy	20 (10 accelerated, 10 standard)	Accelerated speaking valve placement (≤24 h after tracheostomy) vs standard placement (≥48 h after tracheostomy)	Feasibility (recruitment, protocol adherence, time separation), speech intelligibility (Sentence Intelligibility Test), quality of life (QOL-MV), safety events, tolerance, decannulation, LOS, mortality	Median time to valve placement: 22 h (accelerated) vs 45.5 h (standard); no aspiration/hypoxemia/safety events attributable to valve; accelerated group tolerated longer valve trials at 2 weeks (65 vs 15 min, p=0.03); more decannulations in accelerated group (7 vs 1); SIT and QOL not significantly different between groups; feasibility demonstrated	Single-centre, small sample, no blinding (except SIT scoring), possible practice effect, floor effect of SIT, limited generalisability
**39**	Wallace et al. (2023)	Narrative review / expert opinion piece	Mechanically ventilated ICU patients with tracheostomy	N/A	Voice restoration techniques (one-way valve with cuff deflation, above cuff vocalisation, leak	N/A (narrative review)	Restoring natural voice improves communication, quality of life, laryngeal function, swallowing, and facilitates	Narrative review / expert opinion — no primary data

					speech, specialist tracheostomy tubes, augmentative and alternative communication)		weaning/decannulation; one-way valve with cuff deflation recommended as first choice; above cuff vocalisation as alternative when cuff deflation delayed	
**40**	Mills et al. (2023)	Narrative review	ICU patients with tracheostomy (ventilated)	N/A	One-way valves (OWV) and above cuff vocalisation (ACV) to restore laryngo-pharyngeal airflow and subglottic pressure	N/A (narrative review)	Early restoration of laryngo-pharyngeal airflow and subglottic pressure (via OWV or ACV) reduces negative impact on communication and swallowing; OWVs safe in ventilated patients; feasibility of early OWV placement (12-24 h) shown; ACV evidence limited and variable	Narrative review — no primary data; much of the evidence discussed is low quality or from small studies
**41**	Svenberg Lind et al. (2025)	Prospective observational cohort study	Critically ill COVID-19 survivors (tracheotomized with open surgical or percutaneous tracheostomy, or long-term intubated ≥14 days) examined ≥12 months after initial intubation	73 (40 open surgical tracheostomy, 24 percutaneous tracheostomy, 9 long-term intubated)	Airway management (tracheostomy vs prolonged intubation), tracheostomy tube size, duration of tracheostomy	Visible laryngotracheal pathologies (laryngeal, tracheal, skin/soft tissue) via laryngotracheoscopy; patient-reported outcomes (Voice Handicap Index-10, Eating Assessment Tool-10, Dyspnea Index)	58% had visible laryngotracheal pathologies; larger tracheostomy tube size and more days with tracheostomy were associated with tracheal and skin/soft tissue pathology ($p<0.05$); VHI-10 correlated with laryngeal pathology; EAT-10 with skin/soft tissue pathology; highest Dyspnea Index in patients with combined laryngeal and tracheal pathology	Single-region, possible selection bias (symptomatic patients more likely to participate), limited sample size, not all survivors examined
**42**	Xie et al. (2025)	Retrospective cohort study	Persons with traumatic cervical spinal cord injury (SCI) who underwent tracheostomy	78 (48 successful decannulation, 30 non-decannulation)	Tracheostomy decannulation (TD) vs non-TD; factors analysed: AIS grade, Charlson comorbidity index (CCI), age, neurological level, pulmonary function	Successful decannulation rate, time to decannulation, factors influencing decannulation success	Decannulation rate 61.5% (median time 93.5 days); AIS A (OR 5.378, $p=0.021$) and higher CCI (OR 1.836, $p=0.003$) were significant risk factors for decannulation failure; PEF in decannulated group was 145.44 L/min (below classical 160 L/min threshold)	Retrospective, single-centre, limited to hospitalisation period; small sample for pulmonary function tests ($n=26$); no post-discharge follow-up

**43**	Grassi et al. (2020)	Prospective multicentre observational cohort study	Mechanically ventilated ICU patients (≥48 h in controlled mode, then switched to assisted ventilation)	62	Controlled mechanical ventilation (CMV) followed by assisted mechanical ventilation (AMV); daily diaphragm ultrasound (thickness, thickening fraction) every 48 h	Change in diaphragm thickness during CMV and AMV; association with clinical factors and outcomes	Diaphragm thickness decreased during CMV (1.84→1.49 mm, p<0.001) and partially restored during AMV (1.49→1.75 mm, p<0.001); thinning >10% during CMV associated with longer CMV duration (10 vs 5 days, p=0.004) and higher PEEP (12.6 vs 10.4 cmH_2O, p=0.034); recovery of thickness >10% during AMV associated with lower respiratory rate (16.7 vs 19.2 bpm, p=0.019) and RSBI (37 vs 44, p=0.029), and higher Pressure Muscle Index (2 vs 0.4, p=0.024); no association with survival or LOS	Observational design, selection bias (only patients switched to AMV), 18% of thickening fraction measurements discarded as unreliable, multiple statistical comparisons
**44**	Burns et al. (2024)	Randomized clinical trial (2×2 factorial design)	Critically ill adults receiving invasive mechanical ventilation for ≥24 h, able to trigger ventilator, FiO_2 ≤70%, PEEP ≤12 cmH_2O (excluded tracheostomy patients)	797 (198 once-daily + pressure-supported, 204 once-daily + T-piece, 195 more frequent + pressure-supported, 200 more frequent + T-piece)	Screening frequency (once-daily vs more frequent [minimum twice daily]) and spontaneous breathing trial (SBT) technique (pressure-supported with PEEP vs T-piece)	Time to successful extubation (unsupported spontaneous breathing sustained ≥48 h after extubation)	No significant main effects for screening frequency (HR 0.88, 95% CI 0.76–1.03, p=0.12) or SBT technique (HR 1.06, 95% CI 0.91–1.23, p=0.45); significant interaction (p=0.009). More frequent screening + pressure-supported SBT increased time to successful extubation vs once-daily + pressure-supported SBT (HR 0.70, 95% CI 0.50–0.96, p=0.02). Once-daily + pressure-supported SBT vs once-daily + T-piece SBT did not significantly reduce time (HR 1.30, 95% CI 0.98–1.70, p=0.08).	Unblinded design; unexpected interaction requiring pairwise contrasts; exclusion of tracheostomy patients and neurological/neurosurgical ICUs limits generalisability; multiple comparisons not adjusted for secondary outcomes
**45**	Liu et al. (2022)	Retrospective cohort study	Patients with acute exacerbation of COPD (AECOPD) receiving invasive	64 (32 SBT group, 32 non-SBT group)	Spontaneous breathing trial (SBT) with T-piece for 60 min	Reintubation within 48 h of extubation, 28-day mortality	No significant differences between groups: reintubation (12.5% vs 15.6%, p=0.821),	Retrospective, single-centre, small sample

			mechanical ventilation for >48 h, meeting weaning criteria		before extubation vs immediate extubation without SBT		28-day mortality (3.1% vs 6.3%, p=0.554); SBT did not affect extubation success or survival in AECOPD patients	size, relatively short follow-up (28 days)
**46**	Vargas et al. (2024)	Prospective multi-center observational cohort study	Critically ill adult ICU patients requiring elective tracheostomy	694	Elective tracheostomy	ICU mortality	ICU mortality 15.8%; age (OR 1.03), SOFA score at tracheostomy day (OR 1.18), and days of endotracheal intubation (OR 1.05) were independent risk factors for ICU mortality; regression tree identified SOFA ≤3 (7.7% mortality), SOFA 3-9 with age >57 (22.6%), SOFA >9 (41.9%); mortality increased to 33.5% at 3 months, 45.3% at 6 months, 55.9% at 12 months; tracheostomized patients less likely discharged home, more likely to hospital/rehabilitation, and had worse quality of life vs non-tracheostomized at follow-up	Single country (Italy), inhomogeneous population, no comparison with non-tracheostomized ICU patients, loss to follow-up (174/694), lack of data on tracheostomy status in non-survivors at follow-up
**47**	Winkler et al. (2025)	Study protocol (longitudinal prospective observational cohort study, mixed-methods)	Adult inpatients (≥18 y) with tracheostomy (various diagnoses: spinal cord injury, neurological diseases, respiratory diseases) in a specialised neurological rehabilitation centre, for whom decannulation is planned and performed at the study centre, able to communicate in German/French/Italian/English	Planned 150	Decannulation process (observational; no assigned intervention)	Rate and type of physical decannulation-related complications and consequences (re-intubation, re-cannulation, death) occurring up to 3 months post-decannulation	N/A (study protocol — no results yet)	Study protocol — no primary data; single-centre; results may not generalise to other settings
**48**	Devaraja et al. (2024)	Retrospective cohort study	Tracheostomized patients weaned off prolonged mechanical ventilation (non-obstructive indications: neurological illness, respiratory insufficiency, poisoning, etc.), with good	48	Simplified decannulation protocol: Part I (office-based flexible laryngotracheoscopy to assess airway patency, vocal cord mobility,	Efficacy (decannulation rate among patients deemed eligible after Part I) and safety/reliability (successful decannulation among	Efficacy 87.5% (42/48 decannulated successfully); safety/reliability 95.45% (42/44 who tolerated capping had successful decannulation without revision within 1 month); neurological illness	Retrospective, single-centre, no control group, selection bias (only patients with good swallowing clinically), no comparison with other assessment methods,

			swallowing function clinically, age ≥15 years, tracheostomy not for upper airway obstruction		and aspiration-free swallowing) + Part II (tracheostomy capping trial)	those who tolerated capping)	as indication significantly reduced decannulation success (p=0.002)	observer variability not assessed
**49**	Ge et al. (2024)	Prospective observational cohort study	Patients with prolonged tracheostomy tube placement, weaned from mechanical ventilation (>48 h), autonomously breathing, with various primary diseases (pulmonary disease, acute brain injury, ventilatory pump failure, thoracoabdominal surgery, multiorgan failure)	193 (105 decannulated, 80 did not meet protocol criteria)	Standardised decannulation protocol using cough flow measured with tracheostomy tube and speaking valve (CF_SV) >100 L/min as criterion for decannulation readiness; cough augmentation techniques for those with low CF_SV	Decannulation success rate (failure defined as reinsertion of tracheostomy tube within 48 h)	Decannulation success rate 98.1% (103/105) with CF_SV >100 L/min; only 2 failures (1.9%) within 48 h; 3 patients required reintubation within 6 months; CF_SV threshold reliable across different primary diseases	Single-centre, cultural context (measurement with tube in place due to patient/family preferences), small sample for some disease subgroups (e.g., multiorgan failure n=1), need multi-centre validation
**50**	Gallice et al. (2024)	Systematic review	Adult patients with acquired brain injuries (traumatic brain injury, stroke, anoxic brain lesions) who underwent tracheostomy and were free from mechanical ventilation	26 studies	Identification of predictive factors for successful decannulation (including neurological status, age, aetiology, swallowing, cough, pulmonary infections, tracheal lesions, early tracheostomy, CIPN/M)	Predictive factors for successful decannulation (or failure, delayed, difficult weaning)	Strongest predictive factors: higher neurological status, traumatic brain injury (vs stroke/anoxic), younger age, effective swallowing, effective cough, absence of pulmonary infections; secondary factors: early tracheostomy, supratentorial lesions, absence of CIPN/M, absence of tracheal lesions	Systematic review — mostly retrospective observational studies, high heterogeneity in outcomes and definitions, variable decannulation protocols, risk of bias in included studies
**51**	Zheng et al. (2024)	Clinical practice guideline / expert consensus	Adult patients with tracheostomy (including those on mechanical ventilation and long-term tracheostomy)	N/A	Management and rehabilitation strategies (pre-operative preparation, post-operative care, airway clearance, humidification, cuff management, tube changes, decannulation criteria, respiratory muscle training, swallowing and speech therapy, physical exercise, traditional	N/A (recommendations for clinical practice)	Recommendations cover tracheostomy indications, complications, technique selection, post-operative care (secretion management, humidification, cuff pressure monitoring), decannulation criteria (consciousness, airway patency, cough effectiveness, swallowing function, spontaneous breathing trial), and multidisciplinary rehabilitation (respiratory muscle training, swallowing	Clinical practice guideline / expert consensus — no primary data; not a systematic review; based on available literature and expert opinion; publication year not specified

					Chinese medicine, ventilatory support)		training, speech valve use, early mobilisation, acupuncture, herbal medicine)	
